# mTOR pathway targeted inhibition via Rapamycin-loaded PLGA nanoparticles for enhanced bladder cancer therapy

**DOI:** 10.1038/s41598-025-06965-z

**Published:** 2025-07-02

**Authors:** Nour-Elhoda El-hefnawy, Magdy M Youssef, Hassan Abol-Enein, Raghda Abo Gabal

**Affiliations:** 1 Department of Chemistry, Biochemistry Division, Faculty of Science, Mansoura University, Mansoura, Egypt; 2 Center of Excellence for Genome and Cancer Research, Urology and Nephrology Center, Mansoura University, Mansoura, 35516 Egypt

**Keywords:** Biochemistry, Biological techniques, Cancer, Drug discovery, Molecular biology, Oncology, Materials science, Nanoscience and technology

## Abstract

Bladder cancer remains a major clinical challenge due to high recurrence rates, metastatic potential, and the development of drug resistance driven by complex gene regulation. Targeting the PI3K/AKT/mTOR pathway is a promising strategy, as its dysregulation promotes tumor growth and survival. Rapamycin, Everolimus, Temsirolimus and Other ATP-competitive inhibitors work by binding to the mTOR protein and preventing it from activating downstream signaling pathways that control cell growth and division. However, the therapeutic potential of Rapamycin, an mTORC1 inhibitor, is limited by poor solubility, low bioavailability, and non-specific distribution. This study explores the use of poly (lactic-co-glycolic acid) nanoparticles to encapsulate Rapamycin for enhanced delivery and controlled release in bladder cancer therapy. Drug release followed the Korsmeyer-Peppas model, indicating sustained release behavior. In vitro cytotoxicity assays demonstrated that Rapa-PLGA NPs significantly reduced the IC50 compared to free Rapamycin in T24 bladder cancer cells. Wound healing assays revealed substantial inhibition of cancer cell migration. Gene expression analysis showed that Rapa-PLGA NPs effectively downregulated mTOR, HIF-α, BCL-2, and ABCC1, while upregulating FOXO1 and MAPK, promoting apoptosis and reducing drug resistance. These findings highlight the potential of Rapa-PLGA NPs to enhance Rapamycin’s therapeutic efficacy by integrating nanotechnology-driven delivery with gene regulatory mechanisms. This nanoparticle-based system presents a promising strategy for improving targeted bladder cancer therapy and overcoming drug resistance, warranting further in vivo investigation.

## Introduction

Bladder cancer (BC) remains a significant global health challenge, ranking as the fourth most common cancer in men, with high morbidity and mortality rates due to recurrence, metastasis, and therapeutic resistance^1^. At diagnosis, approximately 70–75% of patients present with non-muscle invasive bladder cancer (NMIBC), while 20–25% have progressed to muscle-invasive bladder cancer (MIBC), and 5% exhibit metastatic disease^2^. The complex molecular heterogeneity of bladder cancer, including subtype switching during metastasis, often complicates treatment strategies and contributes to variable therapeutic responses^3,4^. A major contributor to bladder cancer progression is the tumor microenvironment (TME), which supports cancer cell proliferation, inhibits apoptosis, and promotes drug resistance^5–7^.

One of the critical pathways implicated in bladder cancer progression and resistance mechanisms is the phosphoinositide 3-kinase (PI3K)/AKT/mammalian target of rapamycin (mTOR) signaling pathway^8,9^. Aberrant activation of mTOR, particularly due to PTEN loss or mutations, has been reported in up to 55% of MIBC cases, promoting cell survival, proliferation, and metastasis^10^. Dysregulation of this pathway, including loss of the tumor suppressor PTEN and hyperactivation of mTOR, is frequently observed in muscle-invasive bladder cancer (MIBC) and correlates with poor clinical outcomes^11^.

Balanced targeting of the Akt-FOXO-mTOR axis is a critical strategy in cancer therapy due to the complex feedback loops and dual roles of this pathway in regulating cell survival, growth, and metabolism^12^. Hyperactivation of the PI3K/Akt/mTOR pathway is common in many cancers, leading to uncontrolled proliferation and resistance to apoptosis, while simultaneously suppressing the tumor-suppressive activity of FOXO transcription factors^13,14^. However, inhibiting mTORC1 alone can relieve negative feedback on Akt^15^paradoxically reactivating survival pathways. Similarly, forced FOXO activation may upregulate growth factor receptors, unintentionally promoting tumor progression. Therefore, effective therapeutic approaches must carefully balance pathway inhibition to suppress mTORC1-driven tumor growth while preventing compensatory Akt activation and maintaining FOXO’s tumor-suppressive functions^16,17^. Therapies, such as dual PI3K/mTOR inhibitors or PI3K inhibitors with controlled FOXO modulation, offer promising avenues to disrupt cancer cell survival mechanisms while minimizing resistance and preserving normal cellular homeostasis^16^.

Rapamycin (Rapa), a selective mTORC1 inhibitor, has demonstrated potential in suppressing tumor growth by modulating downstream effectors such as S6 kinase and 4E-BP1^18–20^. However, its clinical application is hindered by poor water solubility, rapid systemic clearance, and limited tumor targeting, resulting in suboptimal therapeutic efficacy^21^. Additionally, the complex interplay of gene networks, including overexpression of anti-apoptotic (BCL-2) and drug-resistance-related genes (ABCC1), often undermines the effectiveness of Rapamycin contributing to chemoresistance^22^.While rapamycin inhibits S6K1, it does not fully inhibit 4E-BP1 phosphorylation, thus making it ineffective in blocking cap-dependent translation in most cell types^23^. Phosphorylated 4E-BP1 inhibits pro-oncogenic eIF4E. eIF4E-mediated translation is upregulated in tumors, and blocking this pathway may be crucial to preventing tumor growth in specific cancers^24^.On the other hand, the inhibition of mTORC1 may lead to the feedback activation of IGF-IR and AKT, which compromises the anti-cancer effect of rapalogs^25^. Rapalogs have proved more cytostatic than cytotoxic, perhaps because they also only partially block 4E-BP-dependent translation and fail to inhibit the pro-survival pathways regulated by mTORC2–AKT^26^.

Though rapamycin inhibits mTOR with a high specificity, its effectiveness is dose dependent in different contexts. Two mTOR complexes, mTORC1, and mTORC2, have different sensitivities to rapamycin; different doses of rapamycin are needed to suppress mTOR in different cell lines, as well as the phosphorylation of different mTOR substrates, and these properties of rapamycin dosage can be largely attributed to the competition between rapamycin and phosphatidic acid for mTOR^26^. The downregulation of rictor and 4E-BP1 are particularly significant in view of their association with BC invasion and prognosis. 4E-BP1 was found to correlate with the prognosis of MIBC, and its inactivation using specific inhibitors could be a novel approach for treating MIBC. Rictor has been shown to play a vital role in invasive tumor growth and is considered a critical determinant of BC invasion. More importantly, the downstream effectors of the mTOR pathway, such as VEGF, and HIF-1α, which contribute to angiogenesis and tumor cell survival were also suppressed, leading to significant tumor inhibition following the combination treatment^27^. While mTORC1 is considered to be a direct target for rapamycin, other studies have found that prolonged administration of rapamycin will also lead to mTORC2 inhibition^28^.

Recent advances in nanotechnology offer promising solutions to these challenges. Nanoparticle-based delivery of rapamycin using PLGA FDA approved (Rapa-PLGA) is crucial for enhancing mTOR pathway control by enabling prolonged administration, sustained release, improved cellular uptake, and more effective inhibition of both mTORC1 and mTORC2^29^. Nano-systems offer multiple strategies for precise and controlled drug delivery, responding to external stimuli such as temperature, light, or pH changes^30^. Encapsulation of Rapa within PLGA nanoparticles (Rapa-PLGA NPs) protects the drug from premature degradation and facilitates intracellular delivery, thereby maximizing therapeutic concentration at the tumor site. PLGA NPs are not affected by cell membrane-associated efflux transporters since the drug is well encapsulated. These expected that these NPs could escape the efflux action of these transporters and lead to greater cellular uptake than free drug formulations. In addition, once the NPs enter cancer cells, intracellular release of the drug in a controlled manner can preserve the drug effect and improve the therapeutic efficiency^31^. PLGA nanoparticles improve drug delivery by enhancing tumor accumulation through the enhanced permeability and retention (EPR) effect and enabling sustained drug release. The EPR effect allows nanoparticles to passively target tumor tissues, increasing local drug concentration. Meanwhile, the controlled release from PLGA protects drug from rapid degradation, maintaining therapeutic levels longer than the free drug. Recent studies have demonstrated that PLGA nanoparticles exhibit higher encapsulation efficiency, great stability, and more uniform structure compared liposomes and micelles systems^8,32,33^.

**Fig. 1:**
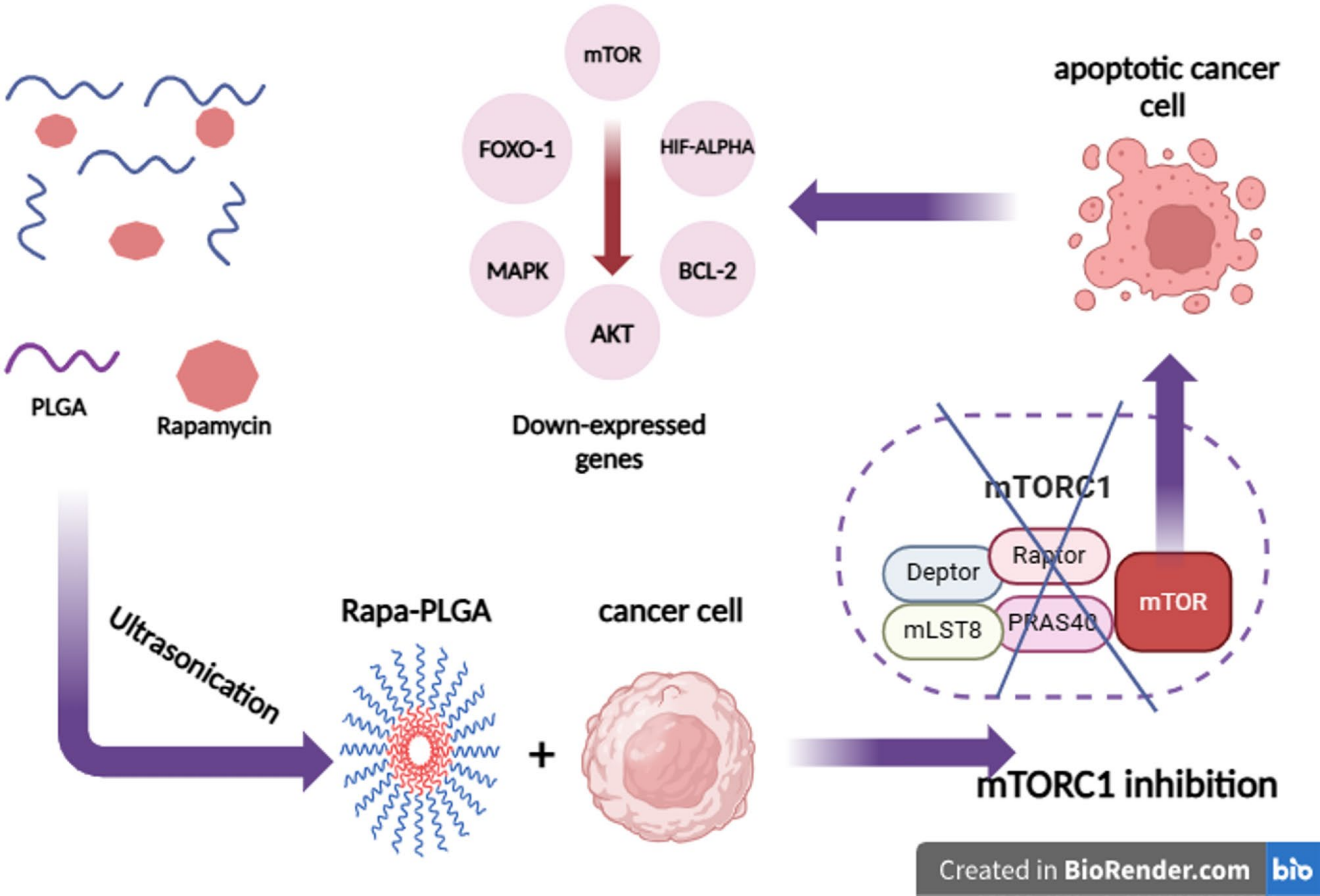
mTOR pathway targeted inhibition via Rapamycin-loaded PLGA nanoparticles.

The novelty of this study lies in the design and application of a PLGA-based nanoformulation of rapamycin (Rapa-PLGA NPs) to enhance intracellular delivery and therapeutic efficacy against muscle-invasive bladder cancer (MIBC). Unlike previous approaches hindered by tumor resistance and limited mTOR inhibition^28^This study utilizes nanotechnology to achieve efficient intracellular delivery and targeted gene modulation as illustrated in Fig. 1. Encapsulation of rapamycin within FDA-approved PLGA nanoparticles enables improved cellular uptake confirmed by transmission electron microscopy (TEM) sustained drug release, and selective tumor cell accumulation. This nano formulation significantly elevated reactive oxygen species (ROS) induced marked mitochondrial damage, and downregulated key pro-survival genes such as BCL-2 and AKT. Moreover, nanoparticle-based delivery systems can modulate gene expression by targeting molecular pathways involved in cancer progression and resistance. By delivering rapamycin directly to cancer cells, Rapa-PLGA NPs effectively downregulated oncogenic genes including mTOR, HIF-α, BCL-2, and ABCC1, while simultaneously upregulating pro-apoptotic and stress response genes such as FOXO1 and MAPK. This dual-action strategy combining enhanced intracellular drug delivery with targeted gene regulation addresses the intrinsic limitations of free rapamycin, offering a more potent and focused therapeutic approach. This study aims to design, synthesize, and characterize Rapa-PLGA NPs and evaluate their effects on gene expression, cytotoxicity, and migration in T24 bladder cancer cells. By integrating nanotechnology with gene-targeted therapy, this work provides a novel and promising strategy for improving bladder cancer treatment and overcoming drug resistance mechanisms.

## Materials and methods

### Materials

Rapamycin (C_51_H_79_NO_13_, ≥ 95%, MW:914.17 g/mol, Sigma-Aldrich, Germany), Poly (D, L-lactide-coglycolide)(PLGA, Actide: Glycolide(50:50), MW:45,000 g/mol, SigmaAldrich, Germany), Dimethylsulfoxide(DMSO, (CH_3_)_2_SO, MW:78.13 g/mol, Sigma-Aldrich, Germany), Polyvinyl alcohol (PVA, (C2H4O)n, MW: 14.000 g/mol, Sigma Aldrich, Mumbai, India).Trypsin(C_35_H_47_N_7_O_10,_ MW: 24,000 g/mol, MP Biomedicals, United States), Dulbecco´s Modified Eagle Medium (DMEM, Capricorn Scientific GmbH, Germany), Fetal Bovine Serum(FBS) (Thermo Fisher Scientific, United States), 3-(4,5-dimethylthiazol-2-yl)-2,5-diphenyl tetrazolium bromide (MTT) was obtained from SERVA, penicillin/streptomycin (100 µg/mL) (Gibco BRL, Grand Island, NY), transzol-up, ROS Modulating 1 ELISA Kit (Nova Lifetech, Cat.NoE1924r), DNA synthesis, the Transcription Kit (Thermofisher, Applied Biosystem), SYBR Green PCR Master Mix (Applied Biosystems, USA), DIETHYLENE TRIAMINE PENTA ACETIC ACID (DTBA, 99%, MW: 393.35 g/mol, LOBA CHEMIE, India).

### Methodology

#### Synthesis of Rapa-PLGA NPs

38.75 mg/ml of Rapa was mixed with 100 mg/ml of PLGA polymer (50:50; Molecular Weight: 40–75 kDa). The resulting organic phases were combined in an ice bath and emulsified by adding 10 mL of 1% w/v polyvinyl alcohol (PVA) and sonicating at 88% power for 10 s using a probe sonicator on ice. To create the secondary water-in-oil-in-water (W/O/W) emulsion, the primary W/O emulsion was added dropwise to a solution containing 0.2% w/v PVA. Homogenization continued for 4 min. The organic solvent was then evaporated using rotary evaporation at room temperature to yield the NP suspension. Purification of the suspended PLGA NPs involved washing in triplicate with Milli-Q^®^ water to remove excess stabilizers and collecting them by centrifugation at 15,000 rpm for 30 min using an ultracentrifuge (Hitachi Koki, Japan)^18^. Liquid was retained for drug analysis and subsequently freeze-dried to further remove any traces of the organic solvent, which was then employed for additional analyses.

### Physical characterization of NPS

#### Transmission electron microscopy (TEM)

CCD camera is linked to Transmission electron microscopy (JEOLJEM-2100, Japan) with a voltage of 200 kV with acceleration. TEM measurements would be done after the samples are diluted 10 times and negatively stained because of the low conductivity of organic samples. The wax plate with a copper grid placement is the first step. Then, a diluted nanosuspension drop would be poured onto the surface of the copper grid for TEM measurements. Moreover, a drop of 2% phosphortungstic acid solution would also be poured onto the wax plate. Finally, the copper grid would be put on the top surface of the spreading phosphor-tungstic acid dispersion for staining for 5 min before TEM measurements^34^.

#### Fourier transform infrared (FTIR)

An FTIR spectrophotometer (PerkinElmer-99075, Germany) is used to study FTIR spectra in the spectrum range of 4000–450 cm^− 1^ with a resolution quality of 4 cm^− 1^ using the conventional KBr pellet approach. By detecting the bonding types, molecular conformations, and functional groups of the active substances in both organic and inorganic compounds, Fourier transform infrared (FT-IR) spectroscopy offers important information on the synthesis process of Rapa-PLGA NPs^18^.

#### UV-Vis spectroscopy analysis for drug loading and encapsulation

The encapsulation efficiency and drug loading capacity of Rapa within the PLGA nanoparticles were determined using UV-Visible (UV-Vis) spectroscopy. The absorption spectra were recorded in the wavelength range of 200–900 nm using a UV/Vis spectrophotometer (Pg Instruments, T80+, China). This range allows the detection of characteristic absorption peaks associated with Rapa and the polymer matrix. In this spectral range, molecules undergo electronic transitions as bonding and non-bonding electrons (n-electrons) absorb energy and are excited to higher anti-bonding molecular orbitals. The characteristic absorbance peak of Rapa was identified and used as an indicator for quantification. A standard calibration curve was generated by measuring the absorbance of known concentrations of Rapa dissolved in DMSO. After nanoparticle preparation, the unencapsulated free drug was separated by centrifugation, and the supernatant was analyzed for residual Rapa content. The amount of encapsulated Rapa was calculated by subtracting the free drug from the initial drug amount^35^. Rapa’s loading efficiency (LE) and loading capacity (LC) were calculated using Eqs. (1), (2) Respectively:

1 $$\:\text{L}\text{o}\text{a}\text{d}\text{i}\text{n}\text{g}\:\text{e}\text{f}\text{f}\text{i}\text{c}\text{i}\text{e}\text{n}\text{c}\text{y}\:\left(\text{L}\text{E}\right)\:\text{\%}\:=\frac{\:\left(amount\:of\:loaded\:Rapa\:in\:mg\right)\:}{\left(amount\:of\:Rapa\:added\:in\:mg\right)}\ast 100$$

2 $$\:\text{l}\text{o}\text{a}\text{d}\text{i}\text{n}\text{g}\:\text{c}\text{a}\text{p}\text{a}\text{c}\text{i}\text{t}\text{y}\left(\text{L}\text{C}\right)\:\text{\%}\:=\:\frac{\left(amount\:of\:loaded\:Rapa\:in\:mg\right)}{\left(amount\:of\:loaded\:Rapa\:in\:mg\right)+\left(amount\:of\:polymer\:in\:mg\right)}\ast 100\:$$

#### In vitro release of Rapa from Rapa-PLGA NPs

In Vitro, the release of Rapa from Rapa-PLGA NPs was conducted using dialysis tubes. Briefly, 2 mL of concentrated Rapa-PLGA NPs suspension (14 mg/ ml) was sealed in dialysis tubes (MWCO = 3.5 KDa) and immersed in 20 mL of PBS solution (pH = 7.4) for neutral and acidic solutions (pH = 6.4), respectively, in an incubator shaker at 37 °C. At predetermined time intervals, 1 mL of release medium in each vial was taken and compensated with another 1 mL of release medium every 2 h for 24 h^36^. After generating a standard curve, the concentration of the drug released from each sample was quantified using UV-Vis spectroscopy at 211 nm, with measurements performed in triplicate. The release kinetics were analyzed using four of the most commonly applied models for drug release from polymeric systems: Zero-Order Kinetics, First-Order Kinetics, the Higuchi Model, and the Korsmeyer-Peppas Model^37,38^.

#### Zeta potential and particle size

The zeta potential(ZP) and the particle size of Free PLGA, and Rapa-PLGA NPs was measured at 25 °C using a zeta potential analyzer particle sizing system (Malvern Zetasize Nano-zs 90, USA). The scattering angle was 90°. Before measurements, the dilution process for the PLGA-encapsulated nanoparticle suspensions was done using deionized water. During a zeta potential analysis, charged colloidal dispersions are placed into a zeta cell (glass cuvette with a round apart). Charged particles would exhibit distinct electrokinetic effects when exposed to an electric field, such as electrophoresis, electroosmosis, streaming potential, or sedimentation potential, which uses the electrophoresis principle to determine zeta potential^39^. The PDI and ZP of Rapa-PLGA NPs stored at 25 °C ± 5 °C were analyzed after 0, 30, 60, 90, 120, 150 days to assess the formulation stability.

#### X-ray diffraction (XRD)

Using an X’PERTPRO diffractometer (Cu Target, λ = 1.54 Å, 45 kV, 40 mA, Hollanda), X-ray diffraction (XRD) analysis was performed. The detection of the XRD patterns was done in the 4°–100° range with a scanning rate of 0.02° min^− 1^. For the fixation of PLGA encapsulated nanoparticles, dried powder on a sample holder, Smooth double-sided sticky tape was used^40^.

#### Hemolysis assay

Blood samples from healthy volunteers were diluted with 10 mL of phosphate buffer saline (PBS), PH = 7.4, and the red blood cells (RBCs) were isolated from the serum using centrifugation at 1200 rpm for 10 min. The RBCs were washed five times and subsequently resuspended in 10 mL of PBS. Subsequently, 200 µL of the RBCs was added to 800 µL of PBS (to serve as a negative control), 0.1% wt Sodium Dodecyl Sulfate (SDS) as a denaturation agent (as a positive control), and different concentrations of Rapa NPs sample (800, 625, 375, 165 µg/mL). The mixtures were incubated at 37 °C for 4 h, next the mixture was centrifuged at 12,000 rpm for five minutes. The supernatants from all samples were collected, and the absorbance was measured at 541 nm using a UV-VIS spectrophotometer (NanoDrop 2000c). The hemolysis ratio (Hr%) in the red blood cells was subsequently determined using the Eq. (3)^41^.

3 $$\:Hr\%=\frac{{A}_{sample}-{A}_{negative\:control}}{{A}_{Positive\:control\:}-{A}_{negative\:control}}\ast 100$$

### Cellular experiments

#### Sample preparation and dose detection

A stock solution of 40 mM of both Rapa was obtained from Sigma-Aldrich and (5mM) Rapa-PLGA NPs, dissolved in DMSO, and kept at 20 °C. human bladder cancer cell line T24 was used in all experiments. The cells were obtained from ATCC and sourced through Nawah Scientific Company. Cells were maintained in DMEM supplemented with 10% fetal bovine serum (FBS) (Thermo Fisher Scientific, United States) and 1% penicillin-streptomycin (Gibco BRL, Grand Island, NY), incubated at 37 °C in a humidified atmosphere with 5% CO_2_. Experiments were conducted using cells at the passage13 and at approximately 70–80% confluency. Cells were seeded at a density of 20 × 10^3^ [cells/cm^2^] and allowed to adhere for 24 h prior to treatments or assays.

#### Cell counting

Trypsin-EDTA (MP Biomedicals, United States) was used to trypsinize the cells, and the cells were then resuspended in an equivalent volume of new media. A 1.5 ml Eppendorf tube was filled with a suspension of 10 µl of cells. After adding 10 µl of 0.4% Trypan blue to the Eppendorf, it was stirred carefully. The coverslip was damp with water, and the hemocytometer was cleaned. A 10× magnification microscope was used to see the chamber on both sides, which was filled with 10 µl of cell suspension. The concentration of viable cells was determined according to Eq. 4

4 $$\:\:\text{A}\text{v}\text{e}\text{r}\text{a}\text{g}\text{e}\:\text{o}\text{f}\:\text{v}\text{i}\text{a}\text{b}\text{l}\text{e}\:\text{c}\text{e}\text{l}\text{l}\:=\frac{total\:no.\:of\:viable\:cells}{total\:no.\:of\:squares\:\ast \:dilution\:factor\:\ast \:{10}^{4}}$$

#### Cell viability assay

A tetrazolium-based test was used to determine cell viability. In short, 96-well culture plates were seeded with 20 × 10^3^ cells per well, and the cells were allowed to grow in media that included 10% fetal bovine serum (FBS). Cells were exposed to varying doses of Rapa and Rapa-PLGA NPs after 24 h. A 0.5% (v/v) dimethyl sulfoxide DMSO negative control was applied. Following a single wash at the designated intervals, cells were treated for four hours at 37 °C with 0.5 mg/mL of 3-(4,5-dimethylthiazol-2-yl)-2,5-diphenyl tetrazolium bromide (MTT). After that, the media was safely disposed of, and the formazan crystals were dissolved by adding (DMSO)^40^. At last, the Microplate Auto reader was used to determine each well’s absorbance at a wavelength of 490 nm (Bio-Tek Instruments Inc., Winooski, VT, USA). Independent experiments were repeated in triplicate, and cell viability was calculated according to Eq. (5).

5 $$\:\mathbf{C}\mathbf{e}\mathbf{l}\mathbf{l}\:\mathbf{v}\mathbf{i}\mathbf{a}\mathbf{b}\mathbf{i}\mathbf{l}\mathbf{i}\mathbf{t}\mathbf{y}\:\left(\mathbf{\%}\right)\:=\frac{\:\:\:\:{\varvec{A}}_{\varvec{s}\varvec{a}\varvec{m}\varvec{p}\varvec{l}\varvec{e}}-{\varvec{A}}_{\varvec{n}\varvec{e}\varvec{g}\varvec{a}\varvec{t}\varvec{i}\varvec{v}\varvec{e}}}{{\varvec{A}}_{\varvec{c}\varvec{o}\varvec{n}\varvec{t}\varvec{r}\varvec{o}\varvec{l}}-{\varvec{A}}_{\varvec{n}\varvec{e}\varvec{g}\varvec{a}\varvec{t}\varvec{i}\varvec{v}\varvec{e}\:}}\ast 100$$

#### In vitro wound healing (scratch) assay

12-well plates were used for seeding T24 cells (20 × 10^3^ cells/well) and attached to their surface with standard incubation conditions for 24 h. scratching the confluent cell monolayers with a sterile plastic pipette tip (200 µl) in a straight line. Rinsing cells with culture medium was an important step to remove free-floating cells and debris. Then, Rapa and Rapa-PLGA NPs were added at final concentrations of (IC50)/well, and the effect on wound healing was monitored. At 24 h, representative scratch zones for every cell line have been captured by the Lecia microscope (Wetzlar, Germany), representative scratch zones for every cell line have been captured by the Lecia microscope (Wetzlar, Germany) a 10 × objective. Each experiment was performed in triplicate for each concentration of Rapa, Rapa-PLGA, and the control. For assessing the results, J image software (Version 1.54 g) was used. For T24 cells, the migration process happened through the scratched area. This was an area where counted within a 400 × 400 μm frame, which was created by the region of interest (ROI) function, making it easy to select 3 random regions in the scratched area. For the T24 cells, the gap between the wound edges was assessed. For both cell lines, three random measurements were made per photographed sample at 24 h, which was used as a baseline. To ignore any differences in environmental conditions in wound healing responses, both cell lines were studied in parallel, and the duration of the microscopic procedure was kept the same environmental condition-related differences in wound healing responses.

The wound healing closure was calculated according to the Eq. 6

6 $$\:\text{W}\text{o}\text{u}\text{n}\text{d}\:\text{C}\text{l}\text{o}\text{s}\text{u}\text{r}\text{e}\text{\%}=\frac{\:\:{\text{I}\text{n}\text{i}\text{t}\text{i}\text{a}\text{l}\:\text{W}\text{o}\text{u}\text{n}\text{d}\:\text{A}\text{r}\text{e}\text{a}}_{\left(0\text{h}\right)}-\:{\text{F}\text{i}\text{n}\text{a}\text{l}\:\text{W}\text{o}\text{u}\text{n}\text{d}\:\text{A}\text{r}\text{e}\text{a}}_{\left(24\text{h}\right)}}{{\text{I}\text{n}\text{i}\text{t}\text{i}\text{a}\text{l}\:\text{W}\text{o}\text{u}\text{n}\text{d}\:\text{A}\text{r}\text{e}\text{a}\:}_{\left(0\text{h}\right)}}\ast 100$$

#### Biological TEM

To improve the contrast of TEM pictures, the cell pellets in the agarose piece underwent standard TEM sample preparation protocol, starting from OsO4 fixation which Cells were treated with OsO4 to increase membrane contrast. Followed by Dehydration & Infiltration serially changing solutions and sticking into LX-112 resin: ethanol: LX-112 (3:1) at RT for 30 min, ethanol: LX-112 (1:1) at RT for 30 min, ethanol: LX-112 (1:3) at RT for 30 min, pure LX-112 at RT for 60 min, twice; 25% ethanol at RT for 15 min, 50% ethanol at RT for 15 min, 75% ethanol at RT for 15 min, 95% ethanol at RT for 15 min, and 100% ethanol at RT for 15 min, two times. Samples were embedded in resin molds and hardened, then Semi-thin sections were cut and stained to locate cells, followed by ultra-thin sections for TEM. Ultra-thin sections were stained with uranyl acetate and lead citrate for contrast. Finally, Sections were imaged using a 200 kV electron microscope. Gatan Digital Micrograph software (Version 2.11.1404.0) was used to acquire and analyze transmission electron microscopy (TEM) images.

#### Reverse transcription polymerase chain reaction

T24 cells were planted in 6-well plates at a density of 20 × 10^3^ cells/well. Every one of the three treatments was applied to the cells: Control, Rapa, Rapa-PLGA NPs for 24 h, and harvested. The whole RNA was extracted by adding 1 ml (Transzol-up plus reagent), then adding 0.2 ml of chloroform, vortexing vigorously for 30 s. Incubate at room temperature for 3 minutes. Centrifuge the tube at 10,000 × g for 15 min at 2–8 °C. A clear upper aqueous phase containing RNA was transferred carefully to a new microcentrifuge tube to avoid DNA contamination. Using an equal volume of absolute ethanol with soft mixing, the mixture was added to a spin column. Centrifugation at 12,000 × g for 30 s at room temperature. Discard the flow-through and washing using CB8 buffer and 500 µl of WB9 buffer, respectively two times. Centrifuge at 12,000 × g for 2 min at room temperature to remove any residual ethanol. For elution, place the spin column into an RNase-free tube. Add 35 µl of RNase-free Water to the center of the column. Centrifuge at 12,000 × g for 1 min at room temperature. For cDNA synthesis, the Transcription Kit (Thermofisher, Applied Biosystem) was used. A reaction mixture of 20 µl using 10X RT Buffer,25X dNTP Mix,10X RT Random Primers, MultiScribe™ Reverse Transcriptase, and RNase Inhibitor.

#### Gene expression assay

Cells treated with IC50 of Rapa, Rapa-PLGA NPs, and control, where cells were collected. RNA was extracted (Transzol up plus), purity and concentration were measured using NanoDrop (Thermo Scientific 2000c). cDNA formation was done using the Transcription Kit (Thermofisher, Applied Biosystem). Real-time PCR for the expression of these genes was detected by SYBR Green PCR Master Mix (Applied Biosystems, USA) using glyceraldehyde 3-phosphate dehydrogenase (GAPDH) as a housekeeping gene. The first step is to denature both strands at 95 °C for 10 min, this process was applied for 40 cycles, 95 °C for 15 s, followed by an annealing process at 60 °C for 1 min, and finally extension at 72 °C for 1 min. These were the cycling conditions for the PCR. The assessment of Data and gene expression calculations was done by (Rotor Gene RT-PCR) according to the 2^−ΔΔCt^ method. Pearson correlation analysis was performed to assess the relationships between mTOR, HIF-alpha, FOXO-1, BCL-2, AKT, ABCC-1, and MAPK gene expressions. The primer sequences listed in Table 1.

**Table 1:** Primer sequence used for gene expression analysis.

Primer name	Sequences
GAPDH	R: TGACCTTGGCCAGGGGTGCT
	F: TGCTGGCGCTGAGTACGTCG
mTOR	R: TTCAGCGATGTCTTGTGAGG
	F: AGTGGACCAGTGGAAACAGG
MAPK	R: AGGACCAGGGGTCAAGAACT
	F: ATCGCCGAAGCACCATTCAA
ABCC-1	R: AGGACACGTCGGAACAAGTC
	F: GGAAGTAGGGCCCAAAGGTC
HIF-α	R: GCTCAGTTAACTTGATCCA
	F: GTGGATTACCACAGCTGA
BCL-2	R: CTTGATTCTGGTGTTTCCC
	F: GTACTTAAAAAATACAACATCAC
AKT	R: CAGGCGACCGCACATCATCT
	F: ACCTTTTGCGGCACACCTGA
FOXO-1	R: AACCTGGCATTACAGTTGGCC
	F: AAATGCAGGAGGCATGACTAC

#### Reactive oxygen species (ROS) assay

The measurement of Reactive Oxygen Species levels were assessed using ROS Modulating 1 ELISA Kit (Nova Lifetech, Cat.NoE1924r) through the sandwich ELISA technique. The preparation of cell lysates was done through rinsing adherent cells using pre-cooled PBS, trypsinization, and centrifugation. Cells were washed, suspended in PBS. The supernatant was used for analysis after centrifugation. 100 µl from a sample, standard, and blank were added to wells for 2 h at 37 °C. Before TMB substrate addition, detection reagents A and B were used for washing. The reaction stopped using sulfuric acid, and absorbance was detected at 450 nm.

### Statistical analysis

GraphPad Prism 8 (GraphPad Software, San Diego, CA, USA) was used to draw the picture and calculate IC_50_ values with experiments performed independently in triplicate. Data are presented as mean ± standard deviation (SD). ImageJ 64-bit software was utilized to quantify wound healing assay results. Gene expression differences among groups were analyzed using one-way ANOVA, followed by Tukey’s post hoc test, performed with SPSS software version 20 (IBM Corp., USA). Statistical significance was set at *p* < 0.05, with *p* < 0.001 indicating a highly significant result and *p* < 0.0001with highest significant. Additionally, Pearson correlation analysis was conducted to explore the relationships between mTOR, HIF-α, FOXO-1, BCL-2, AKT, ABCC-1, and MAPK gene expressions. A heatmap representing both positive and negative correlations was generated using Python’s Seaborn library for data visualization.

## Results and discussion

### Transmission electron microscopy

**Fig. 2:**
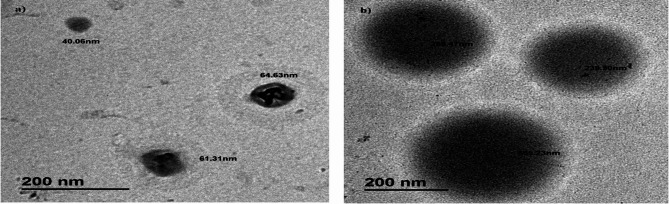
Transmission electron microscopy (TEM) images of functionalized (**a**) free-Rapa, (**b**) Rapa-PLGA NPs showing uniform, smaller particles in the unloaded group and broader, spherical morphology in the drug-loaded group.

Transmission Electron Microscopy (TEM) was employed to examine the size and morphology of both unloaded PLGA nanoparticles (PLGA-free) and drug-loaded Rapa-PLGA nanoparticles as shown in Fig. 2. The TEM images revealed that PLGA-free nanoparticles displayed more uniform and smaller particle sizes, with measured diameters of a mean size of approximately 55.0 ± 12.3 nm. This indicate this narrower distribution and smaller average size in the absence of drug loading. In contrast, Rapa-PLGA NPs exhibited a predominantly spherical morphology with a broader size distribution, ranging from approximately 216 ± 63.35 nm. Notably, the size of Rapa-liposomes was around 100 nm, comparable to the lower end of the Rapa-PLGA nanoparticle size range^8^. The contrast in the images of Rapa-PLGA NPs is sufficient to distinguish particle boundaries, with darker cores and lighter outer regions suggesting a core-shell structure, likely due to PLGA encapsulation. The particles appear well-formed. Particle Size ranges from 100 to 300 nm, are optimal for passive targeting of tumor tissues via the enhanced permeability and retention (EPR) effect. Tumor vasculature is often leaky, allowing nanoparticles of this size to accumulate more readily in tumor tissues compared to larger particles. Additionally, particles under 200 nm can evade rapid clearance by the mononuclear phagocyte system, leading to prolonged circulation time and increased drug accumulation at the tumor site^42^.

### FTIR spectrum

**Fig. 3:**
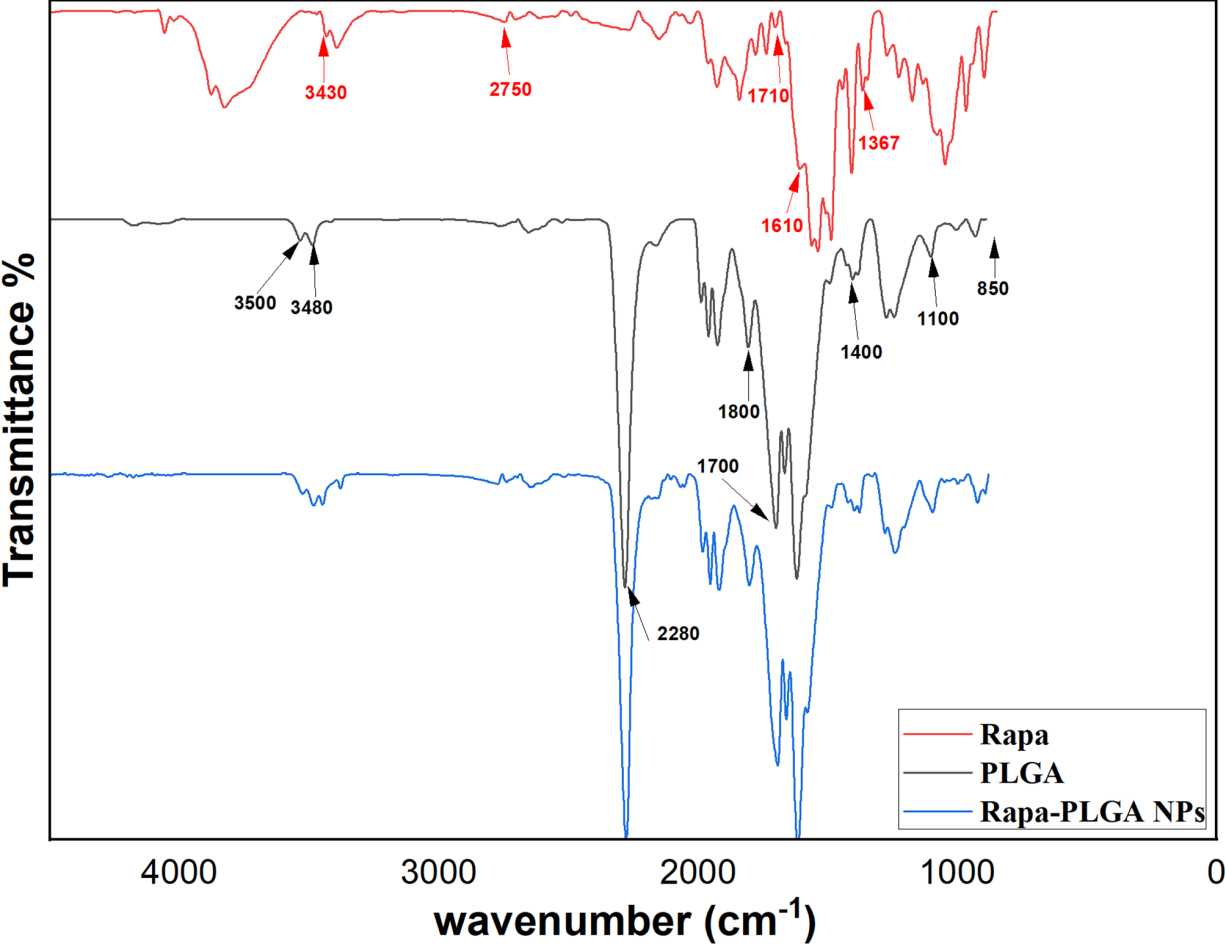
FTIR spectra of functionalized (Rapa, PLGA, and Rapa-PLGA NPs).

The functional groups present in the PLGA/Rapa coating were identified using FTIR analysis. As illustrated in Fig. 3. The PLGA polymer exhibited characteristic peaks, including C–H stretching vibrations at 2970 cm^−1^ and 2918 cm^−1^, and a C = O stretching vibration at 1746 cm^−1^. The C–H bending vibrations appeared at 1461 cm^−1^ and 1398 cm^−1^, while the C–O stretching and C–H rocking vibrations were observed at 1300 cm^−1^ and 1000 cm^−1^, respectively. In addition to these PLGA-specific peaks, Rapa showed distinct O–H stretching at 3473 cm^−1^ and C = C stretching at 1637 cm^−1^^43^. These findings indicate that Rapa retained its chemical stability when incorporated into the PLGA polymer, as no chemical interactions occurred between PLGA and Rapa. The FTIR spectra of the coating displayed all characteristic peaks of both Rapa and PLGA, confirming the preservation of their structures. A comparison of the spectra further supports that Rapa was successfully blended with PLGA without any chemical alteration, consistent with the results reported by Ruixia Hou et al.^43^. The absence of any peak shifts or changes in the spectrum confirms that Rapa was physically encapsulated within the PLGA matrix rather than chemically bonded.

### The Rapa loading efficiency (LE) and loading capacity (LC)

The loading efficiency (LE) and loading capacity (LC) of Rapa in the PLGA nanoparticles were calculated to be 99% and 20%, respectively, based on Eqs. (1) and (2). These values significantly surpass those reported for Rapamycin-loaded liposomes, which showed a drug loading of 9.0% and an encapsulation efficiency of 80%^8^. High loading efficiency is essential, as it directly influences molecular internalization, distribution, and the overall therapeutic potential of Rapa-PLGA nanoparticles. Enhanced loading efficiency suggests increased intracellular drug accumulation following cellular uptake, thereby intensifying the cytotoxic effects on cancer cells. Additionally, it improves nanoparticle biodistribution, promoting targeted delivery to tumor tissues while minimizing exposure to healthy cells. Optimizing drug loading, as demonstrated by these results, is critical for designing nanoparticles capable of effectively delivering Rapa to cancer cells, as prolonged administration is the way to control mTOR pathway in BC^27^.

### In vitro, the release of Rapa from Rapa-PLGA NPs

**Fig. 4:**
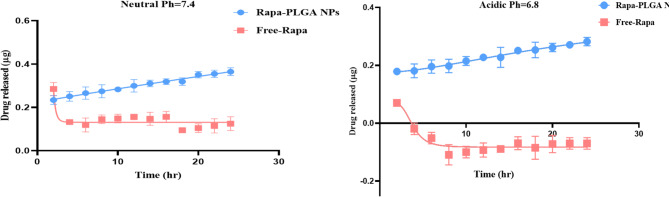
Drug release profiles of Free-Rapa and Rapa-PLGA NPs over 24 h under neutral (pH 7.4) and acidic (pH 6.8) conditions. Free rapamycin shows an initial burst release, while Rapa-PLGA NPs exhibit sustained and controlled drug release.

The drug release profiles of free rapamycin (Free-Rapa) and rapamycin-loaded PLGA nanoparticles (Rapa-PLGA NPs) were evaluated under both neutral (pH 7.4) and acidic (pH 6.8) conditions to simulate physiological and tumor microenvironments, respectively as illustrated in Fig. 4. Under neutral pH, Rapa-PLGA NPs demonstrated a sustained and gradual increase in drug release over time, while free rapamycin exhibited an initial burst release followed by a plateau, indicating limited stability and retention. Similarly, in acidic conditions, Rapa-PLGA NPs maintained a controlled and prolonged release profile, whereas free rapamycin showed an unstable release pattern with an early spike followed by a decline in detectable drug levels.

**Fig. 5:**
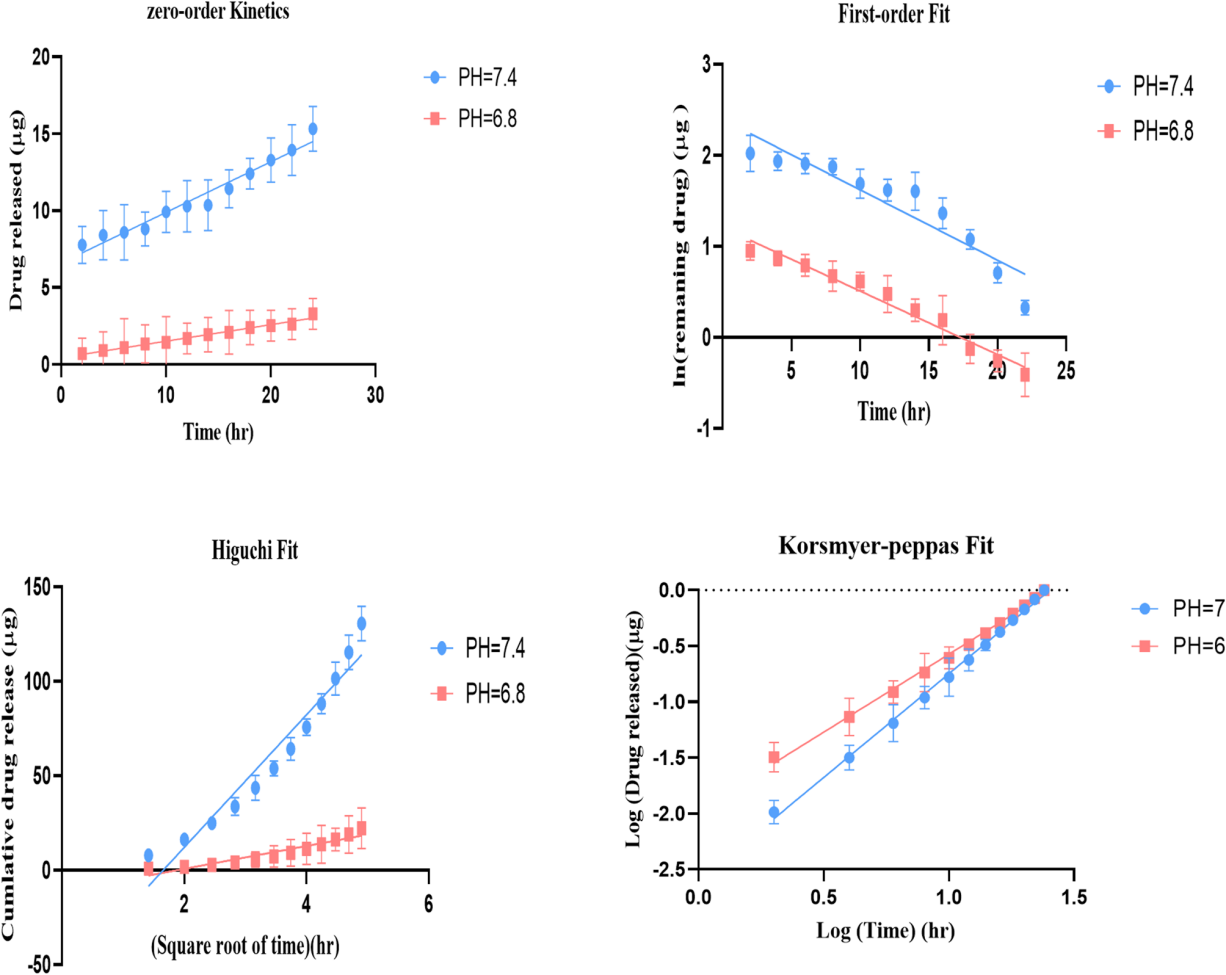
Korsmeyer-Peppas Drug Release Model for Samples: Excellent Fit with R^2^ Values Ranging from 0.99 to 1, Indicating High Correlation Between Experimental and Theoretical Data.

The drug release kinetics of Rapa-PLGA NPs were systematically evaluated by fitting the release data to four mathematical models: zero-order, first-order, Higuchi, and Korsmeyer-Peppas. The fitting parameters and correlation coefficients (R^2^) are summarized in Table 2. At pH 7.4, the Korsmeyer-Peppas model showed the best fit with an R^2^ of 0.99, indicating that the release mechanism is likely governed by anomalous (non-Fickian) diffusion or a combination of diffusion and erosion mechanisms. The zero-order model also provided a good fit (R^2^ = 0.962), suggesting a sustained release profile. However, the first-order model exhibited a lower R^2^ (0.87), indicating less suitability for describing the release under this condition. At pH 6.8, a similar trend was observed where the Korsmeyer-Peppas model exhibited the highest R^2^ value (0.99), confirming controlled and sustained release behavior. Interestingly, the zero-order model showed a slightly higher R^2^ (0.98) compared to the first-order (R^2^ = 0.90) and Higuchi (R^2^ = 0.902) models. These findings suggest that the release of Rapa is better described by diffusion-controlled models with a potential contribution from matrix erosion or polymer relaxation. Further analysis of the Korsmeyer-Peppas model provided mechanistic insights into the release process, as shown in Fig. 5. The Korsmeyer-Peppas plot illustrates the logarithmic drug release profile of Rapa-PLGA NPs at pH 7.4 and pH 6.8. The slope differences between the two curves indicate distinct release mechanisms and rates under different pH conditions. At pH 7.4, the release rate is relatively faster, as reflected by the higher cumulative release over time. The drug release kinetics of two formulations were evaluated using the Korsmeyer–Peppas model, and the corresponding parameters are summarized in Table 2 The fit parameters for different mathematical models (zero-order, first-order, Higuchi, and Korsmeyer-Peppas models) used to analyze drug release from Rapa-PLGA NPs. At pH 7.4 exhibited a release rate constant (k_k_) of 0.08 and a release exponent (n) of 0.881, with a high correlation coefficient (R^2^ = 0.99), indicating a strong fit to the model. The n value suggests an anomalous (non-Fickian) transport mechanism, where both diffusion and polymer relaxation govern the release. In comparison, pH 6.8 had a lower k_k_ value of 0.01 and an n of 0.723, also with an excellent model fit (R^2^ = 0.99). This indicates a slower release rate and a mechanism still consistent with anomalous transport but more diffusion-dominated. The results in Table 2 The fit parameters for different mathematical models (zero-order, first-order, Higuchi, and Korsmeyer-Peppas models) used to analyze drug release from Rapa-PLGA NPs highlight the influence of formulation or environmental conditions on the drug release behavior.

Upon reaching acidic regions, the nanoparticles undergo a charge conversion to positive, promoting cellular uptake due to the negatively charged cell Surface^44^. For theranostics, this charge-switching behavior enables pH-triggered drug release, targeted cellular uptake, and adaptive imaging contrast. In acidic tumor microenvironments or endosomal compartments, the shift in surface charge can enhance nanoparticle–cell interactions through charge-mediated endocytosis, promoting internalization^45–47^. At physiological pH (7.4), Rapa-PLGA nanoparticles carry a negative surface charge due to the deprotonated carboxyl (–COO⁻) groups on PLGA, which leads to electrostatic repulsion with the negatively charged cell membranes of normal cells, limiting cellular uptake. In contrast, under the mildly acidic conditions typical of the tumor microenvironment and the intracellular compartments such as endosomes and lysosomes, the carboxyl groups become protonated, reducing the surface charge and rendering the nanoparticles more neutral. This charge shift enhances their interaction with the negatively charged tumor cell membranes, facilitating improved cellular uptake and enabling sustained intracellular release of rapamycin. This can be attributed to enhanced hydrolytic degradation of the PLGA matrix at neutral pH, leading to a more pronounced erosion-controlled release behavior.

**Table 2:** The fit parameters for different mathematical models (zero-order, first-order, higuchi, and Korsmeyer-Peppas models) used to analyze drug release from Rapa-PLGA NPs.

Compounds	Zero-order fit			First-order fit			Higuchi fit			Korsmyer-peppas fit		
	k_0 (mg/h)_	intercept	*R* ^2^	k_1_	intercept	*R* ^2^	k_H_	intercept	*R* ^2^	k_K_	*n*	*R* ^2^
Rapa-PLGA PH = 7.4	0.3	6.6	0.962	− 0.17	2.4	0.87	35	− 58	0.941	0.08	0.881	0.99
Rapa-PLGA PH = 6.8	0.01	0.04	0.980	− 0.12	1.2	0.90	6.05	− 11.4	0.902	0.01	0.723	0.99

### Zeta potential and particle size

**Fig. 6:**
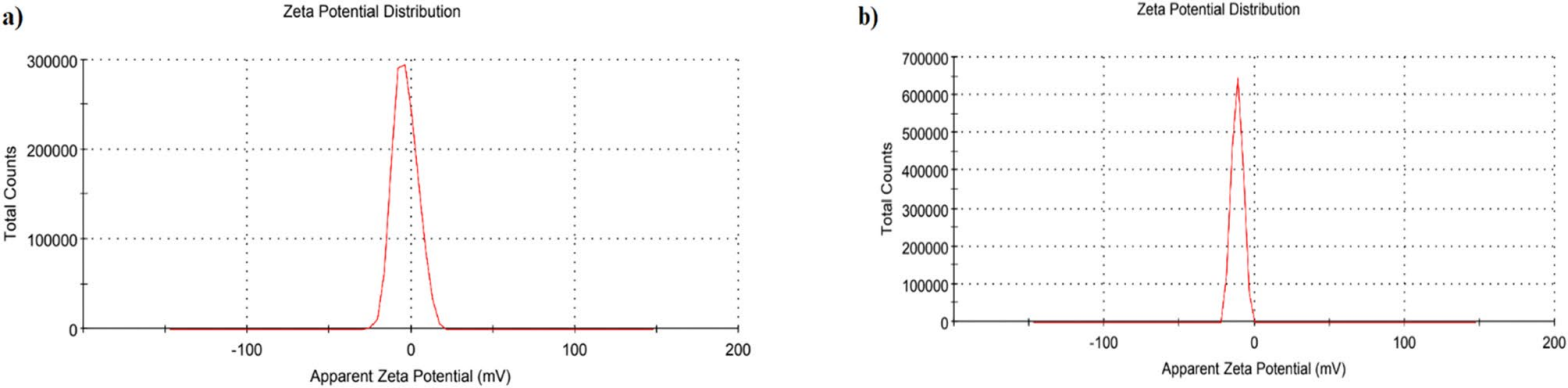
Zeta potential analysis of (**a**) free-PLGA and (**b**) functionalized Apa-PLGA NPs.

The general dividing line of the colloidal system stability can be detected using the zeta potential magnitude. The particles in suspension will repel each other if they have a large zeta potential (negative or positive). Nevertheless, if the particles have low zeta potential values, there is no force to prevent the particles from the electric double layer around the particles. In the current study, dispersion stability is caused by the tight binding of various coating polymers on the surface due to intense intermolecular hydrogen bonding. As shown in Fig. 6, Zeta potential measurements revealed that free PLGA exhibited a surface charge of approximately − 3.8 ± 0.48 mV, indicating limited colloidal stability. In contrast, Rapa-PLGA NPs carried a significantly more negative surface charge of − 11.2 ± 0.45 mV. This increase in negative zeta potential upon loading Rapa onto PLGA suggests enhanced electrostatic repulsion between particles, which contributes to improved colloidal stability and reduced aggregation during storage. In contrast, Rapa-liposomes exhibited a zeta potential of approximately − 3.59 ± 0.72 mV, reflecting a nearly neutral surface charge and reduced colloidal stability^8^.

The therapeutic efficacy of Rapa-loaded PLGA nanoparticles (Rapa-NPs) is significantly influenced by their particle size and zeta potential. Zeta potential indicates the surface charge of nanoparticles, affecting their stability and interaction with biological systems. A zeta potential of − 11.2 ± 0.45 mV suggests moderate colloidal stability; while not highly charged, it is sufficient to prevent immediate aggregation under physiological conditions.

The surface charge of nanoparticles, as indicated by their zeta potential, plays a crucial role in their interaction with cell membranes and, consequently, their therapeutic efficacy. Cell membranes typically possess a net negative charge due to the presence of phospholipids and glycoproteins. Nanoparticles with a positive surface charge can interact more readily with these negatively charged membranes, leading to enhanced cellular uptake. This interaction is primarily due to electrostatic attractions, which can facilitate the adsorption of nanoparticles onto the cell surface and promote endocytosis. However, it’s important to note that while positively charged nanoparticles may exhibit higher uptake, they can also induce greater cytotoxicity, potentially disrupting membrane integrity and leading to cell damage.

Conversely, nanoparticles with a negative surface charge, such as those with a zeta potential of − 11.2 ± 0.45 mV, experience electrostatic repulsion from the similarly charged cell membranes. This repulsion can result in reduced cellular uptake compared to their positively charged counterparts. However, negatively charged nanoparticles often exhibit lower cytotoxicity and better biocompatibility, making them suitable for applications where minimal membrane disruption is desired^48^. In the context of Rapamycin-loaded PLGA nanoparticles with a zeta potential of − 11.2 ± 0.45 mV, the moderate negative charge suggests a balance between stability and biocompatibility. While the negative charge may lead to reduced interaction with cell membranes, it also minimizes potential cytotoxic effects, which is advantageous for sustained drug delivery applications.

**Fig. 7:**
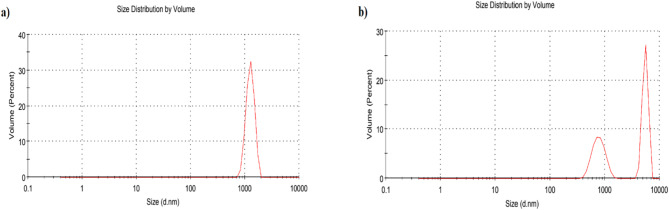
DLS analysis of (**a**) free-Rapa (**b**) and Rapa-PLGA illustrating the size distribution.

Dynamic Light Scattering (DLS) was used to analyze the size distribution by volume for two samples: free -Rapa and Rapa-PLGA NPs, as shown in Fig. 7a,b. DLS measures the fluctuations in light scattering caused by the Brownian motion of particles in suspension, allowing calculation of their hydrodynamic diameter he effective size of the particle including any surface coatings or solvent layers. In Fig. 7a, corresponding to free Rapa, the DLS profile shows a single, sharp peak centered around ~ 1000 nm, indicating a monodisperse but aggregated population. In contrast, Fig. 7b Rapa-PLGA NPs displays a bimodal distribution, with peaks around 300–500 nm and 8000–9000 nm, suggesting a mixture of nanoparticles and larger aggregates. The smaller peak likely represents the primary Rapa-PLGA nanoparticles, while the larger peak indicates particle agglomeration or instability in suspension. The presence of both peaks results in a broader size distribution, a common observation in complex nanocarrier systems. These DLS results differ from those observed by Transmission Electron Microscopy (TEM), which showed much smaller particle sizes (~ 216 nm for Rapa-PLGA) because TEM measures the dry core size of nanoparticles without the surrounding hydration shell or aggregates that influence DLS measurements.

**Fig. 8:**
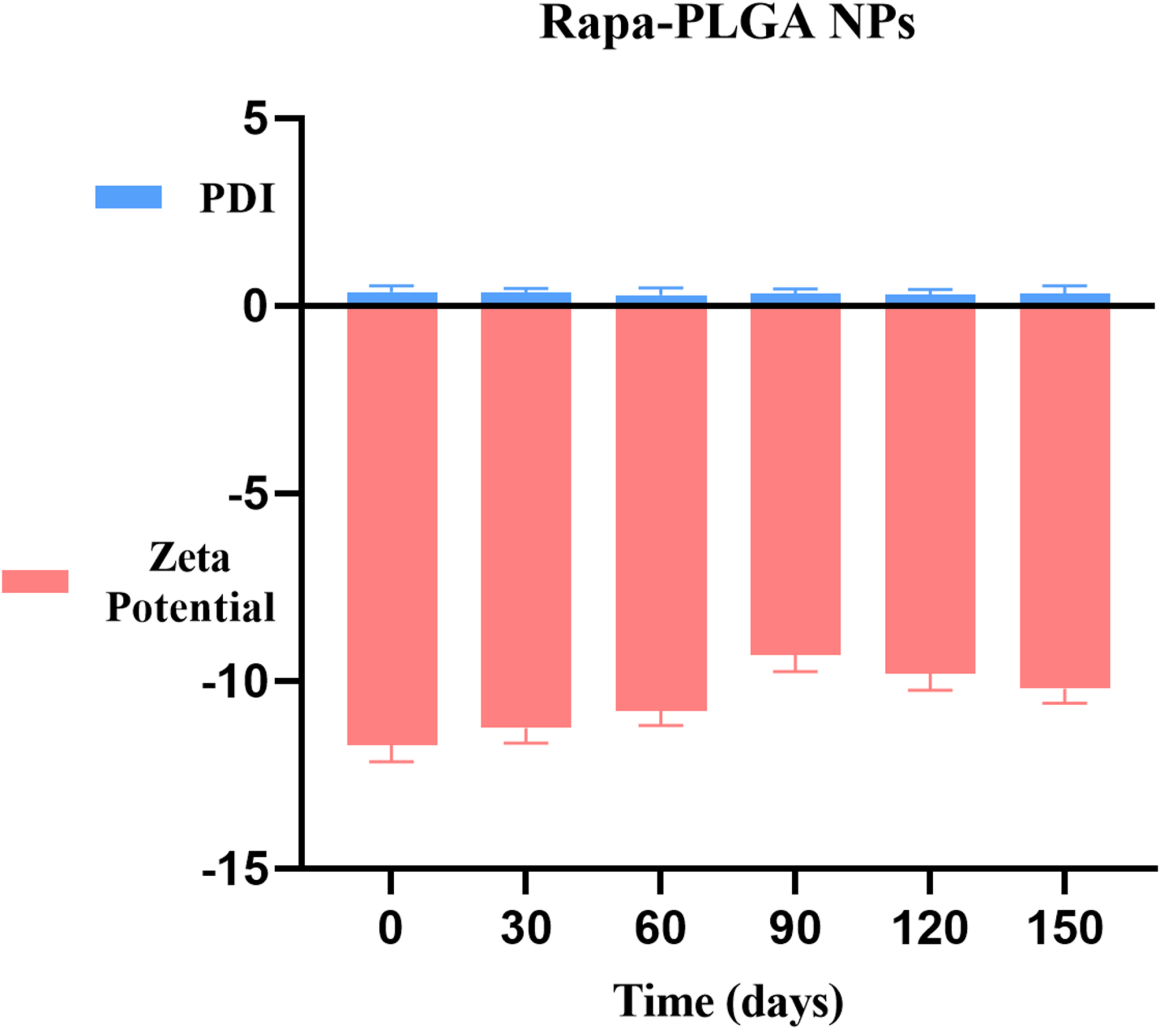
Evolution of the main physicochemical parameters (PDI and zeta potential) of Rapa-PLGA NPs stored in an aqueous medium for 150 days at 25 °C ± 5 °C. Data are presented as mean ± SD with **p < 0.0001.

The stability profile of Rapa-PLGA nanoparticles (NPs) was evaluated by monitoring their polydispersity index (PDI) and zeta potential over a 150-day period in an aqueous medium at 25 ± 5 °C. As shown in Fig. 8, the zeta potential remained consistently negative, ranging from approximately − 10 ± 0.38 mV to − 12 ± 0.5 mV throughout the study. This stable negative surface charge indicates strong electrostatic repulsion between particles, which helps to prevent aggregation and supports colloidal stability. Additionally, the PDI values for free-Rapa (1 ± 0.001), for (0.73 ± 0.1) Rapa-PLGA NPs remained very low and showed minimal variation over time, reflecting a uniform particle size distribution and the absence of significant particle aggregation or degradation. Collectively, these results demonstrate that Rapa-PLGA NPs exhibit excellent physicochemical stability under ambient storage conditions, reinforcing their potential for long-term use in drug delivery applications.

### X-ray diffraction (XRD)

**Fig. 9:**
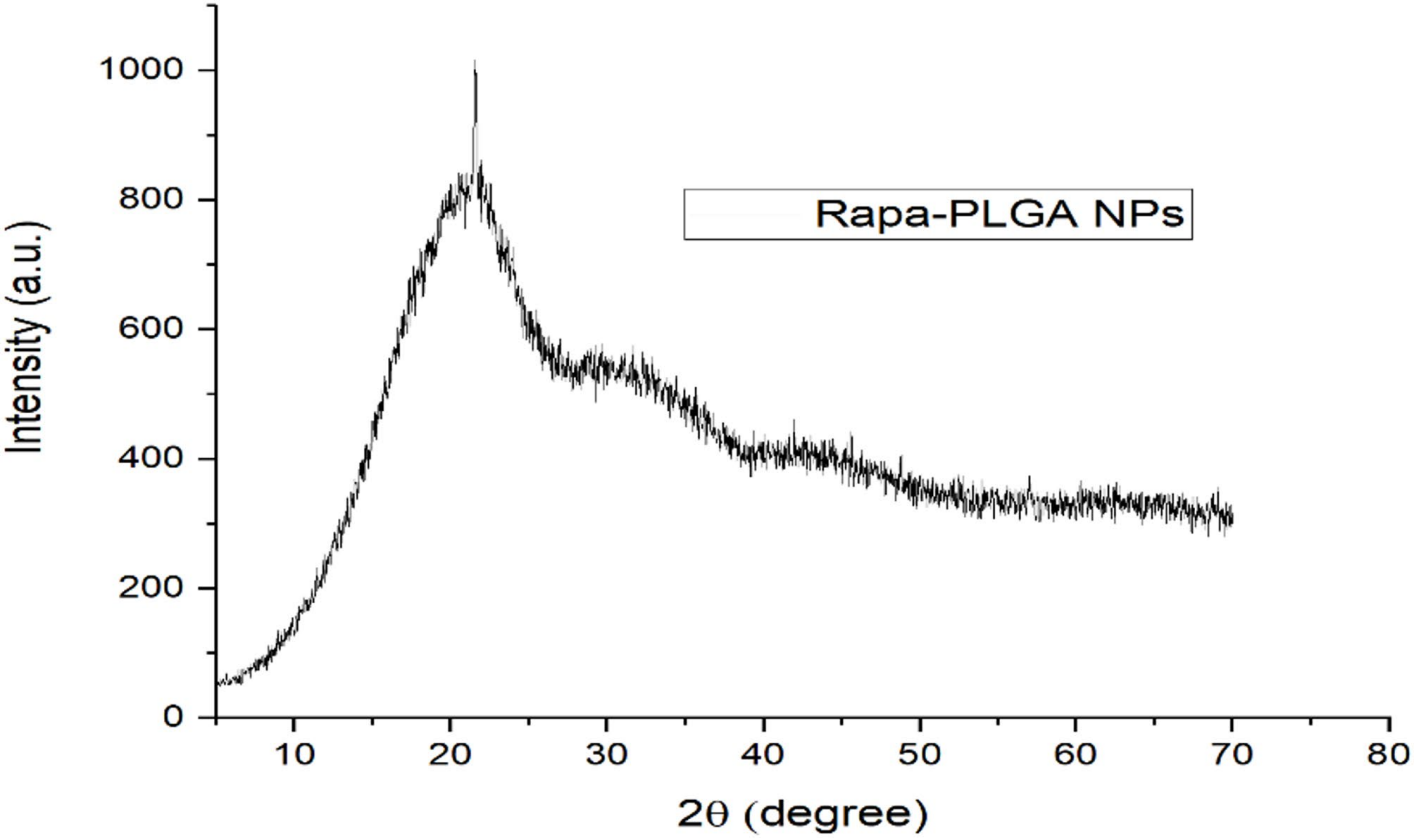
X-ray diffraction (XRD) patterns of functionalized Rapa-PLGA NPs (details).

The X-ray diffraction pattern of Rapa-PLGA NPs exhibit amorphous nature, suggesting that Rapa is well dispersed through the PLGA matrix (non-crystalline-form) (Fig. 9). However, the presence of minor sharp peaks at θ = 20° indicated a small fraction of drug in a crystalline form. The amorphous dispersion of Rapa in the PLGA matrix is beneficial for enhancing drug solubility that increases drug efficacy.

### Hemolysis assay

**Fig. 10:**
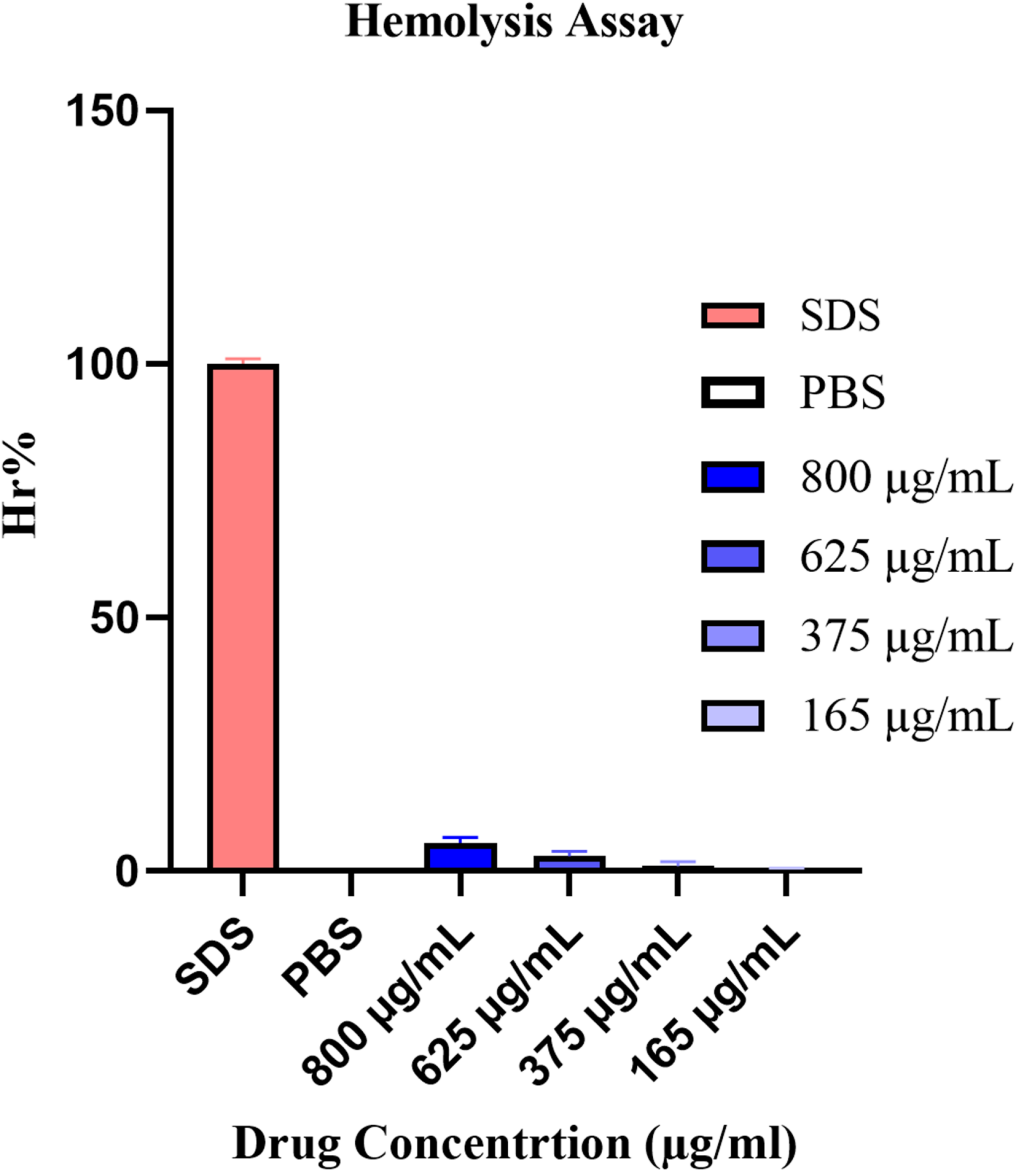
(RBCs)were incubated for 4 h with Rapa-PLGA NPs at different concentrations (800 − 165) µg/mL resulted in blood hemolysis. SDS regarded as positive control whereas PBS was used as negative control.

The hemolysis assay was conducted to evaluate the concentration-dependent effects of Rapa-PLGA NPs (165–800 µg/mL) on red blood cells (RBCs), as illustrated in Fig. 10. Sodium dodecyl sulfate (SDS) served as the positive control, inducing 100% hemolysis, while phosphate-buffered saline (PBS) was used as the negative control, showing minimal hemolysis. A clear concentration-dependent trend was observed, with higher concentrations of Rapa-PLGA NPs, such as 800 µg/mL, inducing greater hemolysis compared to lower concentrations like 165 µg/mL. Despite this trend, the hemolysis levels remained within acceptable biocompatibility limits, supporting the conclusion that Rapa-PLGA NPs are compatible with human blood, in agreement with previous reports^49^.

### Cytotoxicity assay (MTT assay)

**Fig. 11:**
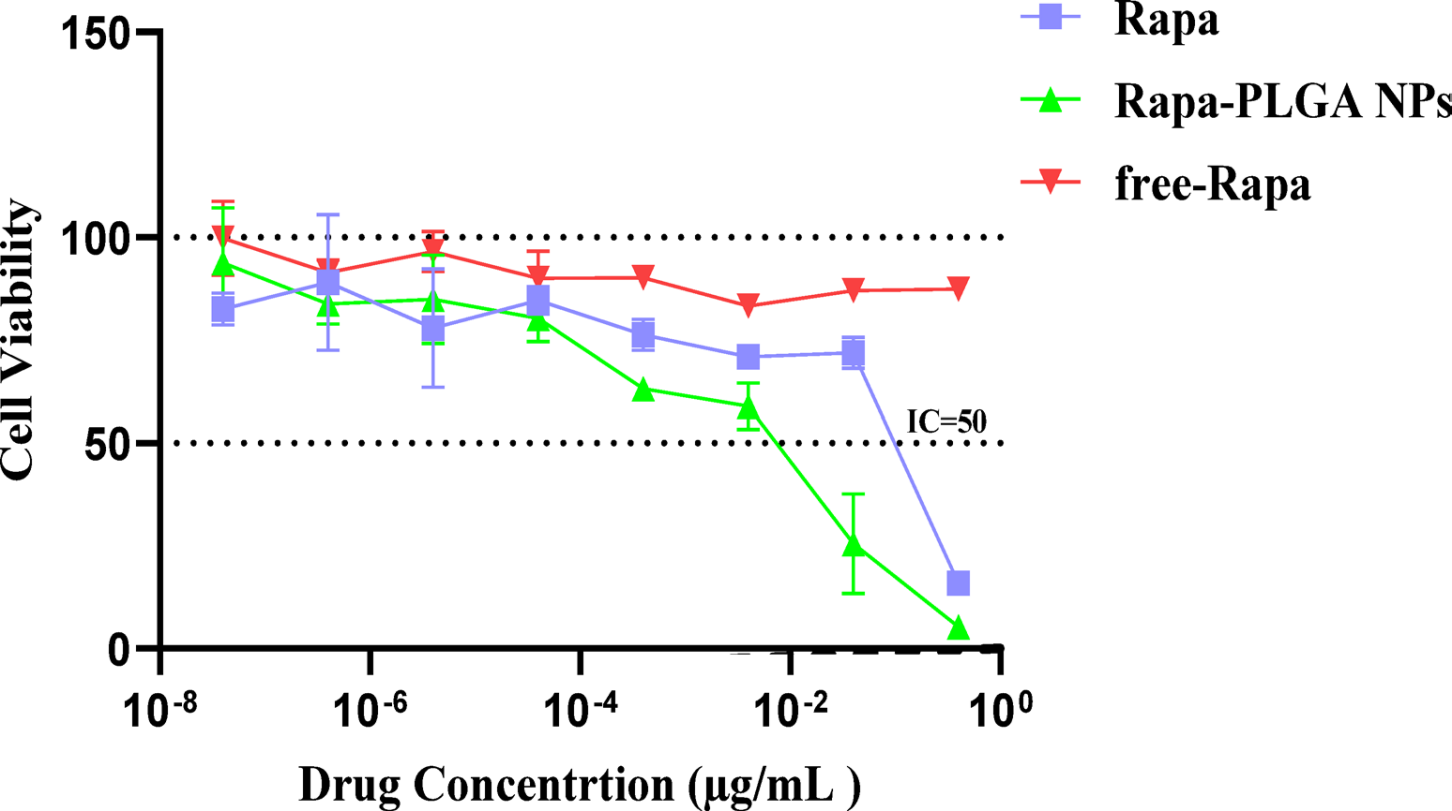
The inhibition of T24 cell growth was evaluated using the MTT assay after 24 h of treatment with Rapa and Rapa-PLGA nanoparticles.

The cell proliferation and cytotoxicity of synthesized Rapa and Rapa-PLGA nanoparticles (NPs) against T24 cells were evaluated using the MTT assay. The results showed that pure Rapa exhibited an IC50 value of 10 ± 0.5 µM, while the IC50 significantly decreased to 1 ± 0.1 µM when Rapa was delivered via PLGA nanoparticles. This substantial reduction in IC50 indicates that the Rapa-PLGA NPs enhanced the cytotoxic effect on T24 cells, demonstrating their potential as an effective drug delivery system for cancer therapy as shown in Fig. 11. Notably, PLGA alone exhibited no cytotoxic effect on T24 cells, confirming its biocompatibility and suitability as a safe and effective drug delivery carrier^50^.

### Wound healing assay

**Fig. 12:**
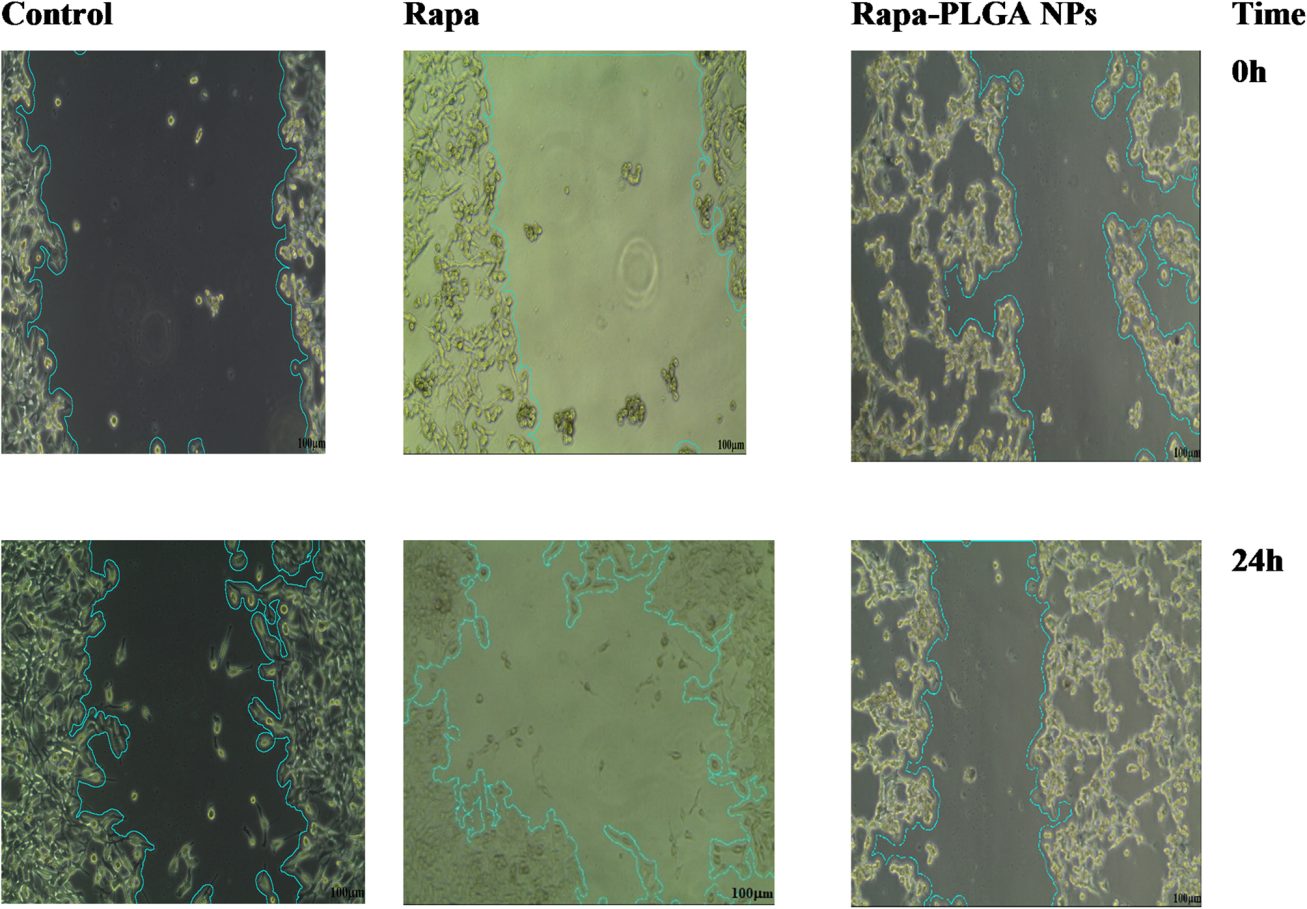
Wound healing percentage at 0 h and 24 h in control, Rapa, and Rapa-PLGA NPs treatment groups. The control group shows substantial natural wound closure over 24 h, while both Rapa and Rapa-PLGA NPs treatments exhibit marked inhibition of the healing process, image analyzed by J image.

In the wound healing study as shown in Table 3, Wound Closure Rates for Control, Rapamy, Rapa-PLGA NPs, the control group demonstrated a wound closure rate of 16.8%, representing the baseline level of natural wound healing within the observed timeframe. This indicates that, without any treatment, wounds were able to close to a certain extent through the body’s natural healing processes. In contrast, treatment with Rapa alone led to a significantly lower wound closure rate of 2.08%. Rapa, a known mTOR inhibitor, is recognized for its anti-proliferative effects, which can inhibit cell growth and migration, key processes required for wound healing. This result suggests that Rapa may slow down wound closure due to its interference with cellular mechanisms essential for tissue repair. When Rapa was formulated as a nanoparticle (Rapa-PLGA NPs), wound healing was even further reduced, with only 1.4% closure observed. The nano-formulated version likely enhances or extends the inhibitory effects of Rapa on mTOR signaling, possibly due to increased stability, targeted delivery, or sustained release, as shown in Fig. 12. These characteristics may prolong the bioavailability of Rapa, leading to a more pronounced suppression of the cellular activities needed for wound repair. Overall, both Rapa and Rapa-PLGA NPs significantly hinder wound healing compared to the control. This observation aligns with the anti-proliferative properties of Rapa, making it potentially useful in cancer treatments where slowing down cellular growth and migration could be beneficial in inhibiting tumor progression. However, in contexts where promoting wound healing is desired, these treatments would likely be counterproductive due to their inhibitory effects on cell proliferation and migration.

**Table 3:** Wound healing assay results: wound closure rates for control, rapa, Rapa-PLGA with statistical significance *p < 0.05.

Sample	Wound area (0 h)	Wound area (24 h)	% wound closure
Control	1130.06 ± 58.9*	940.082 ± 174.2*	16.81%
Rapa	923.06 ± 112*	903.788 ± 106.25*	2.08%
Rapa-PLGA NPs	1364.81 ± 235.5*	1345.02 ± 186.2*	1.20%

### Biological TEM

**Fig. 13:**
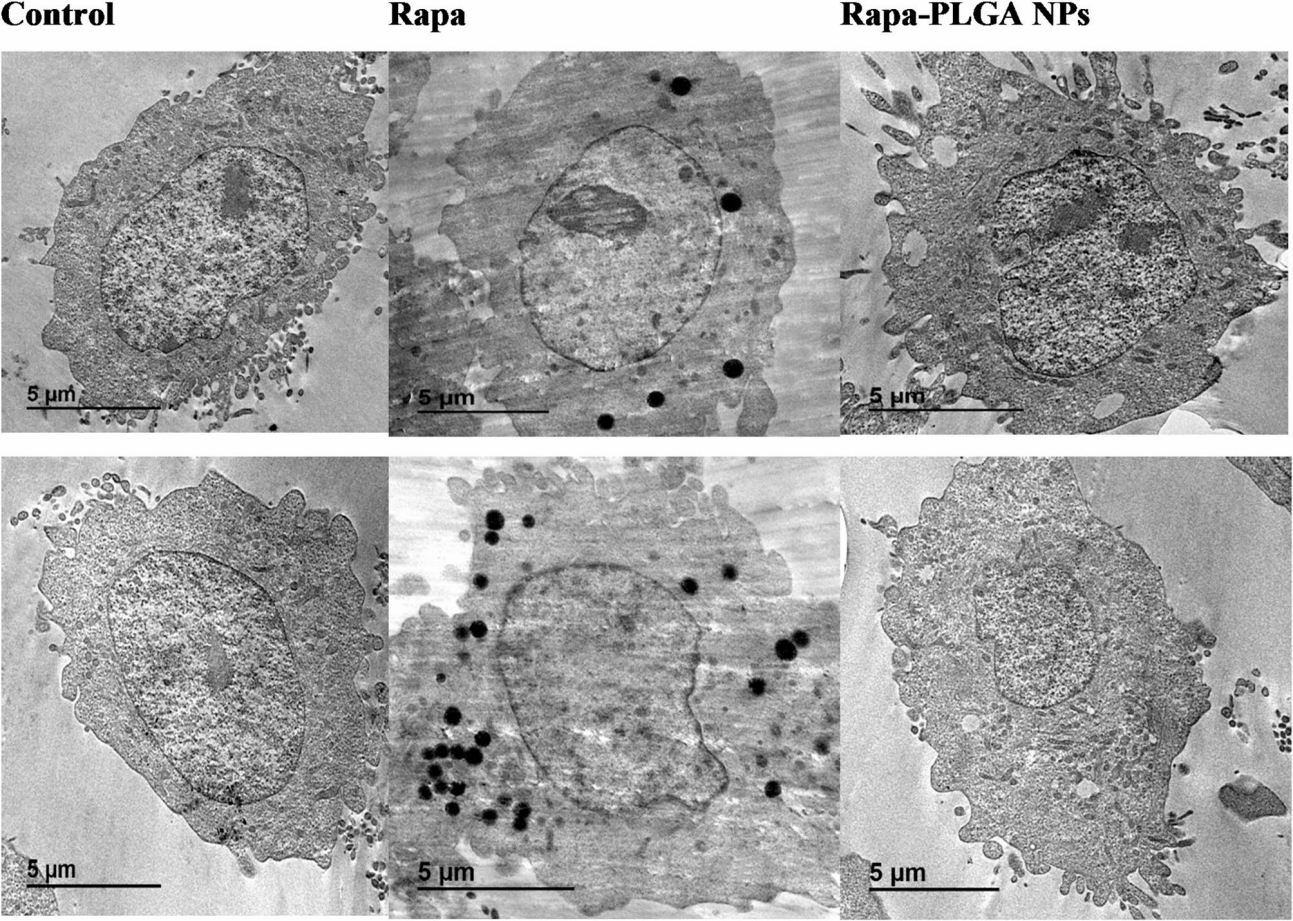
Morphological changes in T24 cells following treatment. Control cells show intact membranes and uniform chromatin. Rapa-treated cells exhibit mild membrane irregularities and chromatin condensation. Rapa-PLGA NPs-treated cells display disrupted membranes, irregular nuclei, and pronounced chromatin condensation.

The comparison of T24 cells across the control, Rapa-treated, and Rapa-PLGA NPs treated groups highlights noticeable differences in cell structure. In the control group, the cell membranes appear intact with minimal irregularities, the nuclei are well-defined, and the chromatin is evenly distributed, suggesting normal cell function. Cells treated with Rapa show subtle signs of stress, including slight membrane irregularities and a shift in chromatin organization, with more condensed areas indicating reduced activity. Meanwhile, cells treated with Rapa-PLGA NPs exhibit more significant changes, such as disrupted membranes, irregular nuclear shapes, and marked chromatin condensation, as shown in Fig. 13.

### Gene expression

**Fig. 14:**
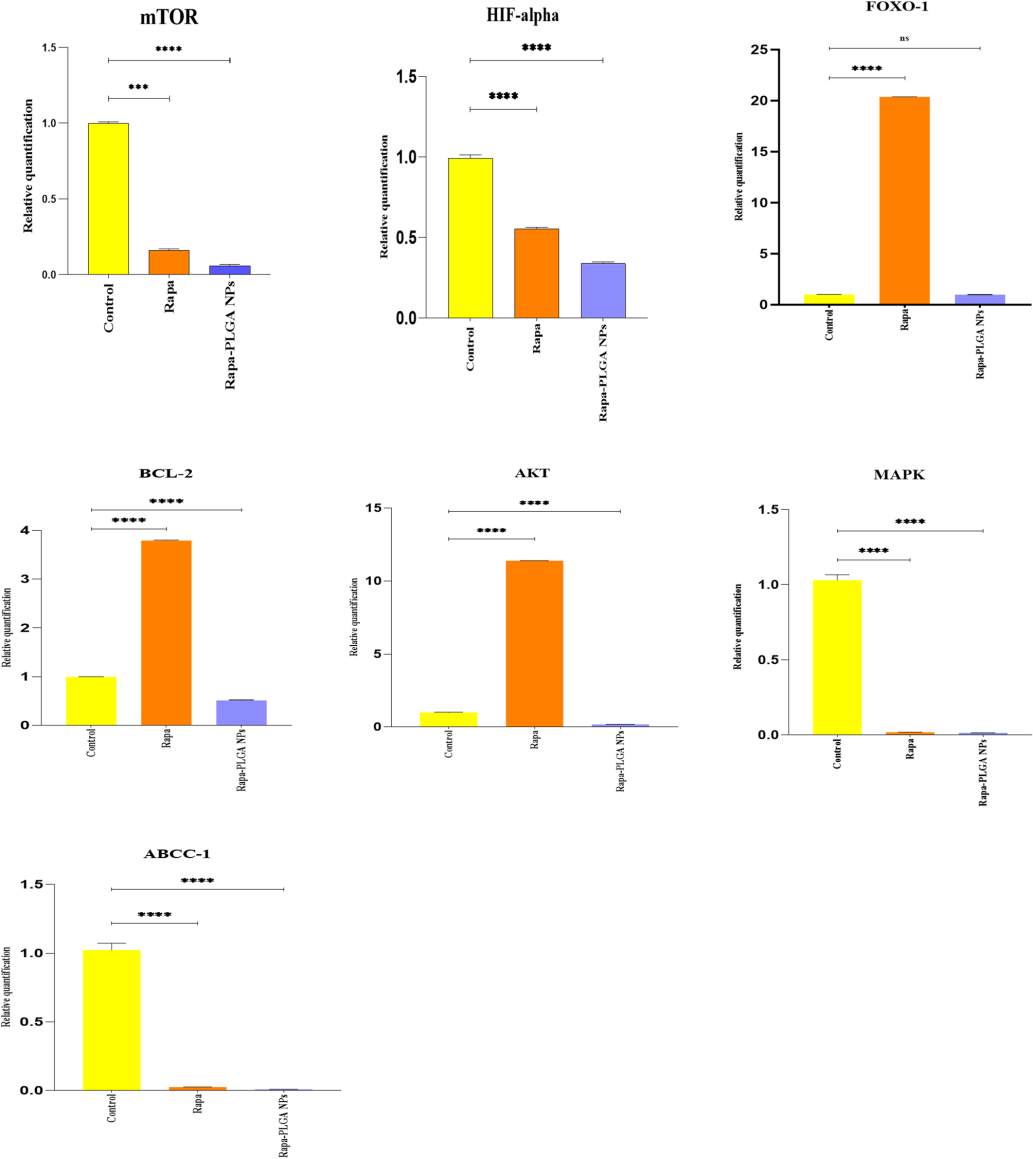
Gene expression of (mTOR, HIF-alpha, BCL-2, AKT, MAPK, FOXO-1, ABCC-1). Comparison between Control(untreated), Rapa, Rapa-PLGA NPs groups.

To evaluate the impact of Rapamycin and Rapamycin-loaded PLGA nanoparticles (Rapa-PLGA NPs) on the Akt-FOXO-mTOR signaling pathway, the study examines how gene expression in bladder cancer cells responds to control and treatments with Free-Rapa, Rapa-PLGA NPs Table 4. This investigation focuses on key genes involved in cell survival, stress response, and drug resistance, providing insights into how these treatments might influence cancer cell behavior and susceptibility to therapy Fig. 14, mTOR, a gene encoding mTOR protein that plays critical role in cell metabolism, growth and autophagy^9^treatment with both Free-Rapa and Rapa-PLGA NPs resulted in significant downregulation of mTOR expression compared to the control group, with the nanoparticles inducing a more pronounced suppression (0.05 ± 0.01 vs. 0.16 ± 0.01; *p* < 0.01). HIF-α, a gene critical for managing low-oxygen (hypoxic) conditions common in tumors, typically aids cancer cells in adapting to hostile environments and resisting^51^. Similarly, HIF-1α, a downstream effector of mTOR, was markedly reduced by both treatments, with Rapa-PLGA NPs exerting a stronger inhibitory effect (0.33 ± 0.01) compared to Free-Rapa (0.55 ± 0.01; *p* < 0.01). These results confirm efficient mTOR pathway suppression, particularly with nanoparticle delivery. This downregulation suggests that these treatments may make cancer cells less adaptable to hypoxia, potentially leaving them more vulnerable to therapeutic interventions. FOXO-1, a gene that induces cell cycle arrest and controls the expression of genes involved in DNA repair, stress resistance, and apoptosis, is just one of the cellular processes that FOXO-1 can affect by acting as an activator or repressor^52,53^. Interestingly, FOXO-1 expression was significantly upregulated following Free- Rapa treatment (20.38 ± 0.01; *p* < 0.01), indicating activation of this transcription factor likely due to feedback regulation upon partial mTOR inhibition. However, Rapa-PLGA NPs maintained FOXO-1 expression at control levels (0.98 ± 0.01; ns), suggesting that robust mTOR suppression by nanoparticles prevented excessive FOXO-1 activation, potentially stabilizing FOXO activity and minimizing uncontrolled feedback loops. AKT, a protein that promotes cell growth and survival, often drives cancer progression when overexpressed. Consistent with the known feedback activation of Akt upon mTORC1 inhibition, free Rapamycin significantly increased AKT expression (11.39 ± 0.009; *p* < 0.01), which goes along with Benjamin et al. study^54^. In contrast, Rapa-PLGA NPs markedly reduced AKT expression (0.17 ± 0.01; *p* < 0.01), indicating superior blockade of survival signaling and effective disruption of the compensatory feedback loop commonly observed with free mTORC1 inhibition, which is confirmed by Chen et al. research^8^. BCL-2, an anti-apoptotic gene, enables cancer cells to evade programmed cell death and thus supports tumor survival^55^. The expression of the anti-apoptotic gene BCL-2 mirrored the Akt activation pattern, showing upregulation in the Rapamycin-treated group (3.79 ± 0.007; *p* < 0.01), while significant downregulation was observed in the Rapa-PLGA NP group (0.51 ± 0.01; *p* < 0.01), suggesting better apoptotic induction with nanoparticle delivery that is confirmed by Song et al. study^56^. ABCC1, a gene linked to drug resistance, helps cancer cells expel chemotherapeutic agents, thereby enabling resistance^57^. MAPK, a gene associated with stress response, growth, and apoptosis pathways^58^. In addition, the drug resistance gene ABCC-1 and MAPK pathway components were substantially reduced by both treatments, with Rapa-PLGA NPs inducing a more pronounced suppression (ABCC-1: 0.008 ± 0.001; MAPK: 0.013 ± 0.001), further supporting enhanced therapeutic potential. The gene expression profile in bladder T24 cells highlights the enhanced inhibitory effect of Rapa-PLGA NPs compared to free Rapamycin. Both treatments significantly suppressed mTOR and HIF-α expression, with Rapa-PLGA NPs showing a stronger effect, suggesting improved inhibition of the mTOR pathway and hypoxia signaling. Interestingly, FOXO-1 expression increased dramatically with free Rapamycin, indicating FOXO activation, while Rapa-PLGA NPs maintained FOXO-1 levels near the control, possibly due to feedback regulation. The pro-survival gene BCL-2 was significantly up-regulated by free Rapamycin but strongly reduced by Rapa-PLGA NPs, implying better apoptotic induction with nanoparticle delivery. Additionally, Akt expression increased sharply with Rapamycin, indicating a compensatory survival mechanism, but was markedly suppressed by Rapa-PLGA NPs, disrupting this feedback loop. Drug resistance gene ABCC-1and MAPK signaling were both significantly downregulated, with greater suppression observed in the nanoparticle group. Overall, Rapa-PLGA NPs demonstrated superior modulation of the Akt-FOXO-mTOR axis, effectively suppressing survival and drug resistance pathways while potentially enhancing therapeutic efficacy in bladder cancer cells.

**Table 4:** Comparison of gene expression in bladder T24 cells treated with rapa, Rapa PLGA NPs.

Gene	Control	Rapa	Rapa-PLGA NPs
mTOR	1 ± 0.01	0.16 ± 0.01*	0.05 ± 0.01**^#^
HIF-alpha	0.99 ± 0.02	0.55 ± 0.01**	0.33 ± 0.01**^#^
FOXO-1	1 ± 0.01	20.38 ± 0.01**	0.98 ± 0.01^ns^
BCL-2	0.99 ± 0.01	3.79 ± 0.007**	0.51 ± 0.01**^#^
AKT	0.98 ± 0.02	11.39 ± 0.009**	0.17 ± 0.01**^#^
ABCC-1	1.02 ± 0.01	0.025 ± 0.001**	0.008 ± 0.001**^#^
MAPK	1 ± 0.03	0.018 ± 0.001**	0.013 ± 0.001**^#^

*p < 0.001 compared to control group, **p < 0.0001 compared to control group, p^#^< 0.0001 compared to Rapa group.

**Fig. 15:**
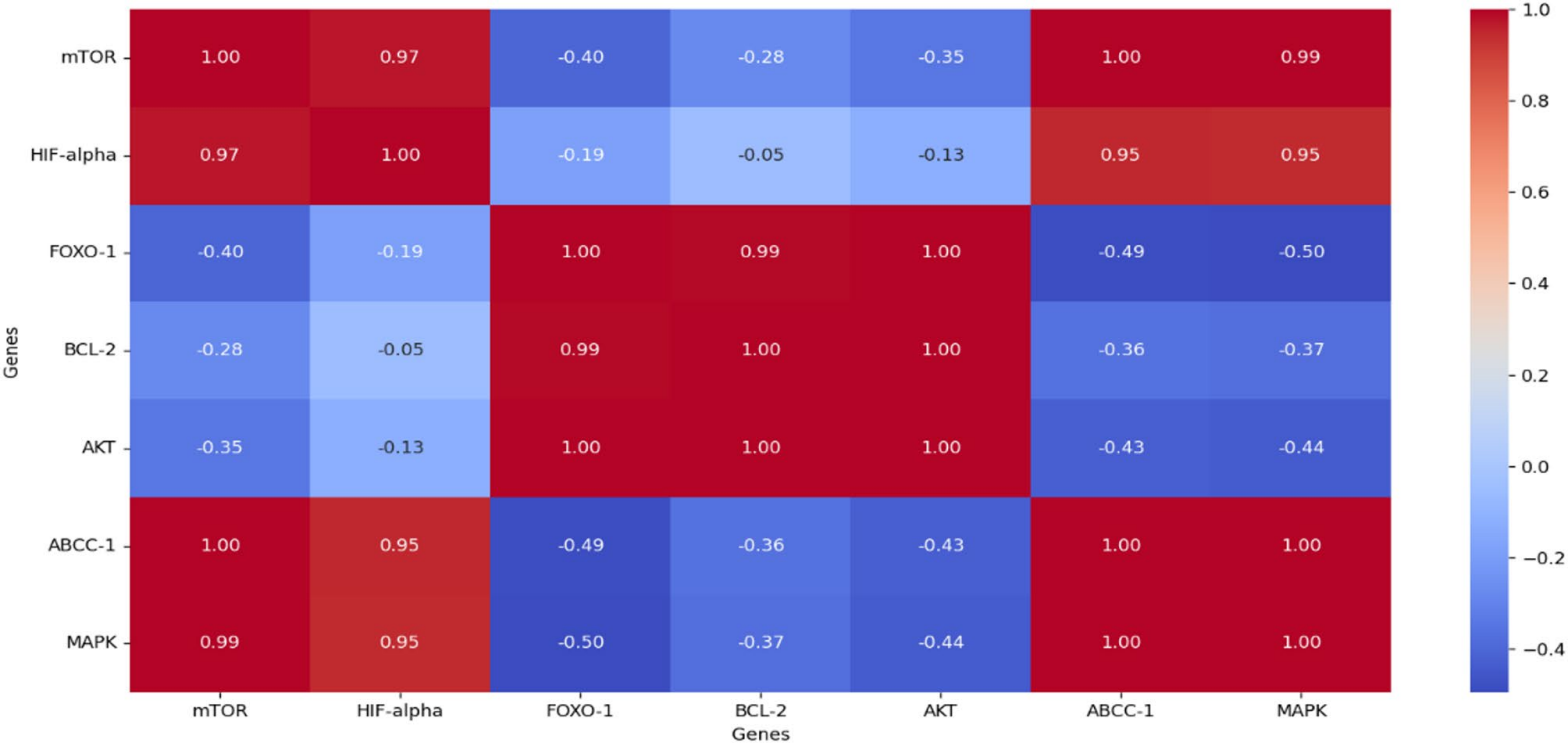
Correlation analysis for interplay between genes (mTOR, HIF-alpha, BCL-2, AKT, MAPK, FOXO-1, ABCC-1), Blue indicates positive correlations, and red indicates negative correlations.

Correlation analysis was performed to further elucidate the interplay between genes involved in the Akt-FOXO-mTOR signaling pathway in bladder T24 cells. The heatmap Fig. 15 Demonstrates strong positive correlations between mTOR, HIF-α, ABCC-1, and MAPK expressions (*r* > 0.95), reflecting the interconnected activation of mTOR-driven survival and drug resistance pathways. Interestingly, FOXO-1 exhibited a negative correlation with m TOR (*r* = − 0.40),HIF-α (*r* = − 0.19), ABCC-1 (*r* = − 0.49), and MAPK (*r* = − 0.50). This inverse relationship supports the concept that FOXO-1 acts as a tumor suppressor, counteracting the pro-survival signaling driven by mTOR and AKT pathways. Additionally, FOXO-1 showed a very strong positive correlation with BCL-2 (*r* = 0.99) and AKT (*r* = 1.00), suggesting complex regulatory feedback, possibly indicating transient FOXO-driven upregulation of BCL-2 under stress conditions. Furthermore, AKT displayed a positive correlation with BCL-2 (*r* = 1.00), reinforcing its role in promoting cell survival. The observed negative correlation between FOXO-1 and survival/drug resistance genes such as ABCC-1 and MAPK further confirms that activating FOXO-1 while suppressing mTOR/AKT may enhance therapeutic efficacy by reducing resistance mechanisms. Overall, these correlations provide additional support for the superior effect of Rapa-PLGA NPs in modulating this axis, favoring apoptotic pathways while suppressing survival and resistance signals.

### Reactive oxygen species assay

The results of the ROS assay demonstrated that The ROS levels measured in T24 bladder cancer cells revealed distinct effects across treatment groups. The control group exhibited a baseline ROS concentration of 25.24 ± 1.15 pg/ml. Treatment with free rapamycin significantly reduced ROS production to 12.64 ± 0.96 pg/ml, indicating an antioxidant or cytoprotective effect, likely due to mTOR pathway inhibition^59^. In contrast, cells treated with rapamycin-loaded PLGA nanoparticles showed a marked increase in ROS levels to 37.12 ± 0.91 pg/ml. This suggests that the nanoparticle formulation either enhances intracellular delivery of Free-Rapa or independently contributes to oxidative stress. The elevated ROS could be indicative of enhanced cellular stress or the initiation of apoptotic pathways, pointing to a potential therapeutic advantage of the nanoparticle system. Further investigation is warranted to delineate whether the observed ROS increase is due to the nanoparticle carrier itself or a synergistic effect with rapamycin shown in Table 5.

**Table 5:** Reactive oxygen species of control, free- rapa, and Rapa-PLGA NPs.

Samples	Reactive oxygen species (ROS) pg/ml
Control	25.24 ± 1.15**
Rapa	12.64 ± 0.96**
Rapa-PLGA NPs	37.12 ± 0.91**

**p < 0.0001.

TEM findings closely align with the measured ROS levels, providing insights into the cellular responses of T24 bladder cancer cells under different treatment conditions. In the control group (ROS: 25.24 pg/ml), cells exhibit intact nuclear morphology and well-preserved cytoplasmic architecture, indicative of normal cellular function and moderate oxidative stress. Treatment with free rapamycin (ROS: 12.64 pg/ml) shows relatively preserved cellular integrity with slight chromatin condensation and fewer ultrastructural abnormalities, supporting the observed reduction in ROS and suggesting a cytoprotective or antioxidant effect of rapamycin^59^. In contrast, cells exposed to rapamycin-loaded PLGA nanoparticles (ROS: 37.12 pg/ml) demonstrate significant morphological damage, including cytoplasmic vacuolization, organelle disruption, and chromatin condensation clear indicators of oxidative stress and early apoptosis. This pronounced ultrastructural degeneration corresponds to the markedly elevated ROS levels, highlighting the potential of the nanoparticle formulation to enhance intracellular stress and trigger therapeutic cytotoxicity. The expression profiles of BCL-2 and AKT in this study align well with their known biological roles in regulating apoptosis and cell survival. BCL-2 is a key anti-apoptotic protein localized primarily to the mitochondrial outer membrane, where it stabilizes mitochondrial integrity and prevents the release of pro-apoptotic factors like cytochrome c. In the rapamycin-PLGA nanoparticle-treated cells, the downregulation of BCL-2 (0.51 ± 0.01) correlates with mitochondrial damage observed by TEM(vacuolization, organelle disruption) and elevated ROS levels (37.12 pg/ml). This suggests loss of mitochondrial integrity, facilitating intrinsic apoptotic signaling consistent with enhanced therapeutic cytotoxicity.

AKT, a serine/threonine kinase, is a key upstream regulator of mTORC2, which in turn activates AKT via Ser473 phosphorylation in a feedback loop. In the rapamycin-only group, the elevated AKT expression (11.39 ± 0.009) reflects a compensatory survival mechanism, likely triggered by partial inhibition of mTORC1 a well-known feedback activation pathway. This aligns with lower ROS production (12.64 pg/ml) and preserved cellular structure in TEM. However, in the rapamycin-PLGA nanoparticle group, AKT expression is dramatically reduced (0.17 ± 0.01), suggesting that the nanoparticle formulation disrupts both mTORC1 and mTORC2 signaling, disabling survival feedback loops and promoting apoptosis. This is supported by the significant downregulation of BCL-2, ROS elevation, and mitochondrial structural collapse, indicating effective inhibition of survival pathways at multiple levels.

## Conclusion

This study demonstrates the successful encapsulation of Rapa into PLGA nanoparticles (Rapa-PLGA NPs) and highlights their enhanced therapeutic potential against bladder cancer. Physicochemical characterization confirmed the stability, sustained release, and improved bioavailability of Rapa-PLGA NPs compared to Free-Rapa. Optimizing nanoparticle size, zeta potential, and sustained drug release is crucial for overcoming the challenges associated with prolonged administration required for effective inhibition of mTOR complex II. In vitro cytotoxicity assays revealed a significant reduction in IC_50_ values with nanoparticle treatment, indicating superior anti-cancer efficacy. Gene expression profiling further supported these findings, showing marked downregulation of mTOR, HIF-α, BCL-2, ABCC1, and AKT alongside modulation of FOXO-1 and MAPK, reflecting effective suppression of survival pathways, drug resistance mechanisms, and enhanced pro-apoptotic activity. Additionally, the wound healing assay demonstrated that Rapa-PLGA NPs significantly inhibited cancer cell migration and proliferation, suggesting potential anti-metastatic effects. Drug release studies revealed that Rapa-PLGA NPs followed the Korsmeyer-Peppas model, indicating a combined diffusion-controlled and polymer relaxation-driven release mechanism under acidic conditions, mimicking the tumor microenvironment. Overall, these findings propose Rapa-PLGA NPs as a promising nanomedicine platform for bladder cancer therapy, offering improved drug delivery, stability, and targeted action. The sustained release of Rapa from Rapa-PLGA NPs in positive way. Future in vivo studies and exploration of combination therapies are warranted to validate their clinical potential and further advance nanoparticle-based cancer therapeutics. Future research should focus on comprehensive pharmacokinetic and pharmacodynamic (PK/PD) studies to better understand the absorption, distribution, metabolism, and excretion profiles of the therapeutic agents used. Additionally, efficacy studies in relevant animal models of bladder cancer are essential to validate the in vivo therapeutic potential and safety of the treatment. These studies will provide critical preclinical data to support translation into clinical applications and optimize dosing regimens for maximal therapeutic benefit.

## Data Availability

All data supporting the findings of this study are available from the corresponding author upon reasonable request. Additional experimental details, raw data, and processed datasets used in the analysis can be provided to interested researchers.
